# Modeling marine microplastic emissions in Life Cycle Assessment: characterization factors for biodegradable polymers and their application in a textile case study

**DOI:** 10.3389/ftox.2025.1494220

**Published:** 2025-03-17

**Authors:** Felicitas Pellengahr, Elena Corella-Puertas, Valérie Mattelin, Nadim Saadi, Francesca Bertella, Anne-Marie Boulay, Yvonne van der Meer

**Affiliations:** ^1^ Aachen Maastricht Institute for Biobased Materials, Maastricht University, Geleen, Netherlands; ^2^ Department of Chemical Engineering, CIRAIG, Polytechnique Montreal, Montreal, QC, Canada; ^3^ Chair of Circular Economy and Sustainability Assessment, TUM Campus Straubing for Biotechnology and Sustainability, Technical University of Munich (TUM), Straubing, Germany; ^4^ Center for Microbial Ecology and Technology (CMET), Ghent University, Ghent, Belgium

**Keywords:** microplastics, life cycle assessment, characterization factor, biodegradation, marine environment

## Abstract

**Introduction:**

With the continuous increase of plastics production, it is imperative to carefully examine their environmental profile through Life Cycle Assessment (LCA). However, current LCA modeling is not considering the potential impacts of plastic emissions on the biosphere. To integrate plastic emissions into LCA, characterization factors are needed that commonly consist of three elements: a fate factor, an exposure factor, and an effect factor. In this context, fate factors quantify the distribution and longevity of plastics in the environment. Research on these fate factors is still limited, especially for biodegradable polymers. Hence, the main objective of this research was to determine the fate factors of biodegradable polymers [poly (lactic acid), poly (butylene succinate), and poly (ε-caprolactam)] based on primary experimental data for the marine environment.

**Methods:**

The validity of former research is tested by comparing the degradation evolution of i. macro- and microplastic particles, ii. two different grades of the polymer, and iii. different temperature levels. The degradation data are obtained by monitoring the oxygen consumption over a period of six months in natural seawater. The determined degradation rates are combined with sedimentation, resuspension, and deep burial rates to obtain fate factors. These fate factors are used to develop polymer-specific characterization factors. The resulting characterization factors are tested in an LCA case study of a synthetic sports shirt made from biodegradable polymer fibers. It allows to assess the relative importance of microplastic impacts compared to other life cycle impacts.

**Results and discussion:**

Comparing the resulting specific surface degradation rates indicates that microplastic degradation rates could be overestimated when using macroplastic degradation data. Pertaining to the case study, the results show that the impact on ecosystem quality by microplastic emissions could account for up to 30% of the total endpoint category. Overall, this work aims to foster interdisciplinary collaboration to leverage the accuracy of LCA studies and thus provide guidance for novel material development.

## 1 Introduction

Clothing and other textiles made from plastic fibers are a significant part of the growing plastic production and its subsequent pollution problem (Henry et al., 2019). It is estimated that the apparel industry contributed to around 14% of the overall plastic pollution in 2019 (Kounina et al., 2024). Introducing biobased and biodegradable plastics not only in the food but also in the apparel industry presents a potential solution to the pollution caused by conventional fossil-based polymers used to date (Friedrich, 2021). Biobased polymers are synthesized either partially or fully from renewable feedstocks of the biosphere (e.g., corn, sugar beet, lignocellulosic biomass), with poly (lactic acid) (PLA) or biobased polyethylene terephthalate (bioPET) being two of the most common examples (Spierling et al., 2018). Biodegradable polymers can be decomposed by naturally occurring microorganisms within a given time frame [depending on the defining standard, e.g., 6 months (ASTM D6691)]. As the biodegradability depends on the presence of suitable microorganisms and hence the receiving environment, biodegradability is a *system property* rather than an intrinsic property (Sander et al., 2024). PLA, e.g., is readily biodegradable in industrial composting (Rosli et al., 2021) but not in seawater (Kliem et al., 2020; Bagheri et al., 2017). Furthermore, biodegradable polymers are not necessarily biobased. Poly (ε-caprolactam) (PCL) for example, belongs to the group of biodegradable, fossil-based polymers (Spierling et al., 2018). Establishing biodegradable plastics for textiles could be a favorable sustainability strategy as those products exhibit unavoidable microplastic emissions to the environment through abrasion during the use phase (Eich et al., 2021). Contrary to microplastics from conventional plastics, those from biodegradable plastics could decompose faster and hence minimize the impact of the microplastic particles (Egan and Salmon, 2022). Although it is commonly believed that biobased products are inherently more environmentally friendly than fossil-based materials, this statement requires a more in-depth examination, such as through the application of the Life Cycle Assessment (LCA) methodology (Mirabella et al., 2013; Moussa et al., 2016). The term “biobased” does not always guarantee environmental friendliness, and this is especially relevant in impact categories that receive less attention than climate change, such as eutrophication, acidification, land use, and water depletion (Moussa et al., 2016).

Most LCA studies on textiles only include cradle-to-gate stages (Munasinghe et al., 2021), neglecting the critical environmental impacts of gate-to-grave stages (Moazzem et al., 2018). Key issues, like microplastic emissions from synthetic and biodegradable fibers, and their effects in aquatic environments, are missing in the literature (Amicarelli et al., 2022), limiting LCA accuracy. However, with the current strive towards a greater adoption of circular products, implementing diverse strategies for value retention like recycling (Garcia-Saravia Ortiz-de-Montellano and van der Meer, 2022), it becomes imperative to consider the impact on the release of microplastics as well.

### 1.1 Plastic emissions in LCA

The current LCA methodology does not adequately address plastic pollution. Life cycle inventory (LCI) modeling has ignored the leakage of plastic into the biosphere and impacts due to this leakage are not comprehensively represented in the life cycle impact assessment (LCIA) methodology. On the LCI side, data and developments from the Plastic Leak Project (PLP) mark the initial steps in assessing plastic leakage (Peano et al., 2020). These efforts have been continued by the Plastic Footprint Network (PFN) which aims to establish harmonized and science-based strategies to assess plastic pollution (Plastic Footprint Network, 2024). The proposed methodology allows to estimate both macro- (greater than 5 mm) and microplastic (smaller than 5 mm) (Woods et al., 2021) leakage for specific plastic applications such as textiles and packaging. Based on the PLP approach, Loubet et al. (2022) proposed a methodology to quantify flows of plastics from the life cycle of seafood products. Regarding the LCIA, comprehensive fate and effect models for plastic pollution are still lacking (Boulay et al., 2021). Efforts to link plastic emissions to LCIA are underway, focusing on developing characterization factors (CFs). Woods et al. (2019) have outlined a preliminary endpoint effect factor for macroplastic entanglement, and Høiberg et al. (2022) proposed an indicator for the potentially affected fraction of species (PAF) due to marine plastic entanglement, applicable in LCIA. Saling et al. (2020) focused on microplastic fate and preliminary eco-toxic effects on marine biota at a midpoint level. Maga et al. (2022) proposed CFs based on plastic’s residence time in the environment, but these lack exposure and effect factors for a complete CF. Corella-Puertas et al. (2023) have developed CFs for physical effects of microplastics in marine environments for eleven polymers. Additionally, Schwarz et al. (2024) evaluated the CFs of microplastics (polypropylene, low-density polyethylene, and PET), using the multimedia fate model Simplebox4Plastics for fate assessment and species sensitivity distributions for ecological effects. Furthermore, macroplastic impacts are included through a conversion fraction. The study integrates these factors into the ReCiPe 2016 method (Schwarz et al., 2024). The inclusion of microplastics as an impact category in LCA is under active methodological development, particularly through the MarILCA (Marine Impacts in Life Cycle Assessment) working group (Boulay et al., 2021), with a primary focus on the marine environment.

Considering the recently proposed methodologies, some LCA case studies have been published that include microplastics. Salieri et al. (2021) introduced a simplified CF for the category freshwater ecotoxicity of 3231 PAF m^3^ *d/kg and applied the result in an LCA study of a polyester T-shirt. Due to the small amount of microplastics released during the production and use phase, the effect of microplastics on overall freshwater ecotoxicity was found to be negligible (Salieri et al., 2021). However, the CF was based on degradation and toxicity data for multiple plastics and was not polymer-specific. A comprehensive overview of other case studies is provided in Table 1.

**TABLE 1:** Overview of LCA case studies including microplastic emissions.

References	Content	LCI	LCIA	Comment
Almeida et al. (2022)	Quantification of plastics emitted to the environment considering a FU of 1 kg of landed octopus	x		Based on Loubet et al., 2022
Boone et al. (2023)	Plastic packaging: lifetime costs on marine ecosystem services	x	x	Impact of leakage based on its potential contribution to marine litter
Del Saavedra Oso et al. (2023)	Assessment of PHA-based packaging	x	x	Based on Corella-Puertas et al., 2022
Galafton et al. (2023)	Plastic pollution as impact category assessing strawberry production	x		Based on Maga et al. (2022), primary release to soil
Sadeleer and Woodhouse (2024)	Environmental impact of mulch film in agricultural application	x		Based on dynamic material flow analysis
Salieri et al. (2021)	Assessment of a polyester T-shirt including plastic emissions to freshwater	x	x	Simplified CF for the category freshwater ecotoxicity
Sanchez-Matos et al. (2024)	Marine plastic (macro and micro) emissions from seafood trade between the European Union and South America	x		Based on Loubet et al., 2022
Schwarz et al. (2024)	Comparative assessment of multilayer packaging including macro- and microplastic loss	x	x	Based on material flow analysis and Simplebox4plastics (Quik et al., 2023)
United Nations Environment Programme (2022)	Single-use food packaging and their physical effects on biota impact	x	x	Based on Corella-Puertas et al. (2022)
Wiedemann et al. (2023)	Accounting for microplastic emissions during use of sweater	x		Based on Peano et al. (2020)
Zeilerbauer et al. (2024)	Comparison natural and artificial turf pitches	x	x	Based on Corella-Puertas et al. (2023)

If the life cycle inventory (LCI) category is fulfilled microplastic losses are quantified. If the Life Cycle Impact Assessment (LCIA) category is selected it indicates that the quantified emissions have also been integrated into the impact assessment (FU, functional unit).

### 1.2 Methodological background

Within the MarILCA framework, physical effects on biota has been proposed as a new midpoint impact category that allows the inclusion of microplastics effects on organisms through external (entanglement, smothering) and internal (ingestion) pathways (Woods et al., 2021). To translate the emission of a substance to its contribution to an impact category, CFs are needed, with the structure shown in Equation 1 for midpoint CFs (Rosenbaum et al., 2008). This structure is commonly used in emission-based LCA, e.g., also for the acidification potential CFs (Roy et al., 2014) or particulate matter formation CFs (Gronlund et al., 2015).

$$\text{Characterization}\;\text{factor}=\text{Fate}\;\text{factor }\left(\text{FF}\right)\times \text{Exposure}\;\text{factor}\times \text{Effect}\;\text{factor}$$ (1)

FFs are depending on multiple factors such as degradation and transport phenomena (e.g., windage, Langmuir cells, sedimentation and resuspension, etc.) which have been thoroughly discussed in previous research (Hajjar et al., 2024). Simplified fate mechanisms have been integrated into FF modeling as first proposed by Maga et al. (2022) and further developed by Corella-Puertas et al. (2023) and Saadi et al.^1^ As the most recent CFs for ocean water and sediments rely on the degradation rate as one of the main components, this research will focus on the degradation of microplastics. However, it should be noted that other environmental conditions affecting the degradation rate will become relevant when including also the coastal compartment (e.g., UV light influence). Several factors influence the biodegradation of plastics. These factors can be distinguished into material characteristics (e.g., chemical structure (Witt et al., 1999), molecular weight and hence the plastic grade (Tokiwa et al., 2009), and molecular orientation (Cho et al., 2003), particle characteristics [specific particle size (César et al., 2009), general shape (Yang et al., 2005), and surface area (Chinaglia et al., 2018)], as well as environmental influences [temperature, water salinity, UV exposure (Ghosh and Jones, 2021)]. Although marine biodegradation has been studied in previous research (Hino et al., 2023), the datasets are often not suitable for CF modeling, e.g., as the dimensions or a precise description of the tested sample are missing. Since current modeling approaches for degradation rates used in fate factors of plastics are based on literature data, assessing the uncertainty of results is difficult.

### 1.3 Research aim

Therefore, the aim of this study is defined through the following goals:

Goal 1 Calculation of specific surface degradation rates (SSDRs; see explanation in Section 2.4) based on experimentally derived data for biodegradable polymers. This includes the validity of CF modeling approaches of former research by testing the main assumptions as listed below.

• Macroplastic degradation results can be applicable to microplastics (assess the effect of size).
• Degradation data derived from incubation at temperatures above seawater temperatures are representative (assess the effect of temperature).
• The same degradation rate can be applied for several grades of one polymer (assess the effect of polymer grade).

Goal 2 Integration of SSDRs into FF and CF modeling and understanding different effects of the calculation model (effect of the initial size, shape, and degradability). The developed CFs are meant to contribute to the literature of CFs for biodegradable polymers in seawater.

Goal 3 Testing the developed CFs in a case study of a sports shirt made from biodegradable polymer fibers. The case study serves only as an illustrative example to showcase the integration of microplastic emissions into product-based LCA studies.

## 2 Materials and methods

To investigate the research aims described above, an integrative approach (employing both experimental research as well as LCA-related modeling) was developed, which entails the methods for the three goals.

1. Preparation of degradation experiments through material selection and classification of samples (Section 2.1 and 2.2; Goal 1)
2. Degradation experiments: Incubation of polymer samples in seawater to obtain the biodegradation rates (Section 2.3; Goal 1)
3. Goal 1: Calculation of the SSDR as proposed in Corella-Puertas et al., 2023 and built on the degradation model of Chamas et al. (2020) (Section 2.4; Goal 1)
4. Combining the SSDRs with sedimentation, resuspension, and deep burial rates to calculate the FF; combining the FF with exposure and effect factors to obtain CFs (Section 2.5 and 2.6; Goal 2)
5. Testing the developed CFs in an LCA case study of a sports shirt, addressing the potential physical effects on biota impacts of biodegradable microplastics and their relative importance compared to other impact categories (Section 2.7; Goal 3)

### 2.1 Materials

For the incubation experiments, three polymers were chosen. To investigate different conditions and effects (Goal 1; temperature, polymer grade, particle size; see Table 2), poly (ε-caprolactam) (PCL) was selected. PCL is a synthetic polymer that is known to be biodegradable in various environments including seawater [e.g., recently demonstrated by Hino et al. (2023)] due to its similarity to the natural polymeric compound cutin (Murphy et al., 1996). Additionally, it is expected that PCL will be available as a biobased polymer in the near future (Maaskant et al., 2023). Furthermore, poly (lactic acid) (PLA) was selected as it is one of the most studied and used biobased polymers. Moreover, to extend the knowledge and database for fate factor modeling, poly (butylene succinate) (PBSA) was included in the study. PCL granulate was purchased from Perstop AB, Malmö, Sweden (as CAPA6250 and CAPA 6500, further denoted as *PCL grade A* and *PCL grade B*, respectively, which differ in molecular weight), PLA 6302D granulate from Total Corbion, Amsterdam, Netherlands (as Luminy L130), and PBSA FD92 granulate from MCC Mitsubishi, Tokyo, Japan.

**TABLE 2:** Overview of incubated samples and differentiating test conditions.

Handle	Polymer	Grade	Shape	Temperature (°C)	Relation to research aim
PCL-A_p_20	PCL	A	Powder	20	Baseline, addition to CF value database
PCL-A_g_20	PCL	A	Granulate	20	Effect of size
PCL-A_p_4	PCL	A	Powder	4	Effect of temperature
PCL-B_p_20	PCL	B	Powder	20	Effect of polymer grade
PLA-p_20	PLA	-	Powder	20	Addition to CF value database, applicability to case study, validation of former research
PBSA-p_20	PBSA	-	Powder	20	Addition to CF value database

The handle in the left column is used for distinction purposes throughout the following, with p indicating a powdered sample, g a granulate sample, A and B the respective grades A and B of PCL, and 20 and 4 the incubation temperatures at 20°C and 4°C, respectively.

### 2.2 Classification of polymer powders

The polymer granulates were cryo-milled (through liquid nitrogen purchased from Linde Gas, Schiedam, Netherlands, and ground in a grinding mill from IKA, Staufen, Germany) as it was demonstrated before that cryogrinding does not affect the chemical prerequisites for biodegradability (Hino et al., 2023). The resulting polymer particles were then morphologically analyzed by employing a Polarized Optical Microscope (POM) to determine their shape and to conduct a statistical study on the particle size distribution. The optical micrographs were taken between cross-polarizers with an Olympus BX53 microscope mounted with an Olympus DP26 camera. The particle dimensions were measured using a tool present in the software OLYMPUS Stream Essentials 2.4, which allowed for the measurement of particle length and width since they were not spherical. The magnification used for this study was ×5 or 10x, as specified in the micrographs.

### 2.3 Degradation experiments

#### 2.3.1 Methods of degradation testing

Each polymer was incubated in freshly sampled seawater (Oostende, Belgium; N 51,2355884; E 2,9137014, sampled on 20/12/2022). The seawater was supplied with additional nutrients, according to the ASTM D 6691 – 09 norm (ASTM, 2018). The nutrients were added to a final concentration of 0.5 g/L NH_4_Cl and 0.1 g/L KH_2_PO_4_. Serum flasks were filled two-thirds with seawater, leaving one-third as headspace (air). The plastic was added in 1 g/L. The flasks were closed air-tight and incubated either at 20°C or at 4°C (see Table 2) in the dark. The 20 °C incubated flasks were shaken at 100 rpm, the 4°C flasks could not be shaken due to practical limitations. The samples were set up in triplicates, except for the abiotic controls. Abiotic controls are necessary to ensure that there is no carbon dioxide producing activity other than the microbial one. This is achieved by adding 160 mM of NaN_3_. The controls with only seawater were set up in triplicate as well. Samples were taken for headspace analysis, flow cytometry, and pH every 2 weeks. The degradation tests were run for a period of 6 months in total.

#### 2.3.2 Calculation of the biodegradation based on CO_2_ evolution

CO_2_ evolution (as suggested by the standard ASTM D 6691 – 09) was preferred over direct mass loss determination as the latter is only an indicator for fragmentation but not necessarily for biodegradation through bacterial activity (Mattelin et al., 2023; Agarwal, 2020). Monitoring the evolving CO_2_ also allows to plot the degradation evolution rather than only the final degradation after the incubation has been completed. It is further assumed that methane emissions can be excluded as the aerobic conditions are maintained during the incubation period (García-Depraect et al., 2022). The biodegradation percentage ( $\text{De}g_{i}\left(t\right)$ , see (Equation 2) of the material i is determined by dividing the mass of CO_2_ produced by the material at a specific time $m_{\text{CO}2,i}\left(t\right)$ (with the blank sample $m_{\text{CO}2,\text{blank}}$ subtracted) by the theoretical CO_2_ amount ( $Th_{\text{CO}2,i}$ ). $Th_{\text{CO}2,i}$ is determined by Equation 3 with $m_{0,i}$ being the initial total polymer mass per sample and $C_{\text{tot}}$ the total carbon content. The factor 44/12 is necessary to approximate the mass of CO_2_ based on the mass of carbon. The theoretical CO_2_ amount would be produced in case of the complete mineralization of the sample (Massardier-Nageotte et al., 2006; Narancic et al., 2018). The presence of an intrinsic imbalance occurs because some carbon is utilized in forming the reacting biomass, which does not convert to CO_2_ and prevents achieving 100% conversion. The amount of biomass was neglected in the carbon balance because it was found by Eubeler that it does not affect the biodegradation (Eubeler, 2010). Generally, this error remains below 5%–10%. The total carbon content for each sample is given in Table 3. Evolved CO_2_ in the pure seawater samples was caused by the intrinsic oxidation activity of the microorganisms present in the inoculum, without the addition of a biodegradable carbon source, i.e., plastic (Eubeler, 2010). Hence, it is important to determine the intrinsic activity to correct the CO_2_ measured from the test assays containing the test substance by deducting the blank values.

$$\text{De}g_{i}\left(t\right)=\frac{m_{\text{CO}2,i}\left(t\right)-m_{\text{CO}2,\text{blank}}\left(t\right)}{Th_{\text{CO}2,i}}$$ (2)

$$Th_{\text{CO}2,i}\left(\text{mg}\right)=m_{0,i}\times C_{\text{tot}}\times \frac{44}{12}$$ (3)

**TABLE 3:** Total organic carbon content of the sampled materials.

Polymer	C_tot_ (%)	References
PCL	45.4	Massardier-Nageotte et al. (2006)
PLA	49.3	Massardier-Nageotte et al. (2006)
PBSA	52	Calculation, this study

### 2.4 Specific surface degradation rate

Following Maga et al. (2022) and Corella-Puertas et al. (2023) which both used the degradation model proposed by Chamas et al. (2020) only surface degradation is assumed to take place, leading to the determination of the specific surface degradation rate (SSDR, v_d_) according to Equation 4 [adapted from Maga et al. (2022)].

$$v_{d}=\frac{1}{2}\frac{d_{0}}{t}\left(1-\sqrt[{a}]{1-\frac{∆m}{m_{0}}}\right)$$ (4)

with the relative mass loss $\frac{∆m}{m_{0}}$ after an incubation time t determined through the degradation experiments, based on the evolved CO_2_. The characteristic length, which is the initial diameter $d_{0}$ of the particles, was evaluated as described in Section 2.2 as well as the parameter a , depending on the shape of the particle. In the present study, a is 3 as the particles showed a spherical shape.

### 2.5 Fate factors

The fate factors of this work describe the fate mechanisms of microplastic particles in the marine ecosystem (i.e., the water column, surface water, and sediment). These fate mechanisms are dominated by degradation and sedimentation (Corella-Puertas et al., 2023). While the degradation rates used in this research are polymer-, size-, and shape-dependent, the sedimentation rates only depend on the density of the polymer (Corella-Puertas et al., 2023). The resulting SSDR was used to determine the degradation rate constants for different microplastic shapes (sphere, fiber, and film) which were combined with sedimentation, resuspension and deep burial rates to develop fate factors for each polymer considering the different shapes and sizes, as described in Saadi et al.^1^ The work of Saadi et al.^1^ is a continuation of Corella-Puertas et al. (2023) by adding impacts in the sediment compartment. The methodology is detailed in Saadi et al. *(under review)* (with the same code, transfer and loss rates being used for this research). Whilst the fate factors as an intermediate step are not discussed hereafter, they are shared in the Supplementary Information (SI) in Supplementary Note S1 for future research purposes.

### 2.6 Characterization factors

The ecosystem level exposure and effect factor (EEF) of Corella-Puertas et al. (2023) for the water column and surface water (EEF_w_), as well as the EEF_sed_ for sediments of Saadi et al.^1^, were used to obtain the characterization factors for physical effects on biota of microplastic emissions at both midpoint (problem) and endpoint (damage) levels for each polymer. The EEF_w_ had been updated from Lavoie et al. (2022) by adapting it to the latest USEtox recommendations and therefore aligning it with GLAM (the UNEP-hosted Global Guidance on Life Cycle Impact Assessment Indicators (GLAM) project), applying EC10 instead of EC50 data. For the sediments, the EEF_sed_ developed by Saadi et al.^1^ was used and is also based on EC10 data. The generic EEFs (water and sediments) were then combined with fate factors computed in this work to obtain midpoints CFs following the methodology of Saadi et al.^1^. Saadi et al.^1^ have scaled compartmental-level impacts (expressed as a Potentially Affected Fraction (PAF) of *water*- or *sediment*-dwelling species) to ecosystem level CFs (PAF of *marine* species) using a feeding exposure compartment approach. It should be noted that the EEFs are for physical effects only, which is the reason why solely pure polymer data were taken into account, excluding polymers with additives. The midpoint CFs resulting from Equation 1 are in line with the ecotoxicity impact category (Fantke et al., 2017) and therefore expressed in Potentially Affected Fraction m^3^/kg_emitted_. The endpoint CFs are determined through Equation 5, expressed in Potentially Disappeared Fraction of species (PDF) m^2^ yr/kg_emitted_, in line with the units of the IMPACT World + methodology (Corella-Puertas et al., 2023).

$$\text{Endpoint CF}=Midpoint\;CF\frac{SF}{Water\;depth}$$ (5)

With SF being the severity factor (in PDF/PAF) and the water depth expressed in [m]. To integrate the endpoint CFs into the case study which used ReCiPe, an additional conversion step was necessary as shown in Equation 6 (the rationale is based on Corella-Puertas et al. (2023) discussed in the Supplementary Note S2).

$$\left[\frac{\text{PDF}\cdot m^{2}\cdot y\;r}{kg_{\text{emitted}}}\right]\cdot 3.46\cdot 10^{-12}\;\frac{species}{m^{3}}\cdot 100\;m\Rightarrow \left[species\cdot yr\right]$$ (6)

### 2.7 Life cycle assessment case study

The developed CFs were tested in an LCA case study of a sports shirt, allowing to identify the magnitude of the potential impact of microplastic emissions compared to established impact categories. The LCA was conducted according to the ISO14040 series (International Organization for Standardization, 2006).

#### 2.7.1 Goal and scope

**FIGURE 1:**
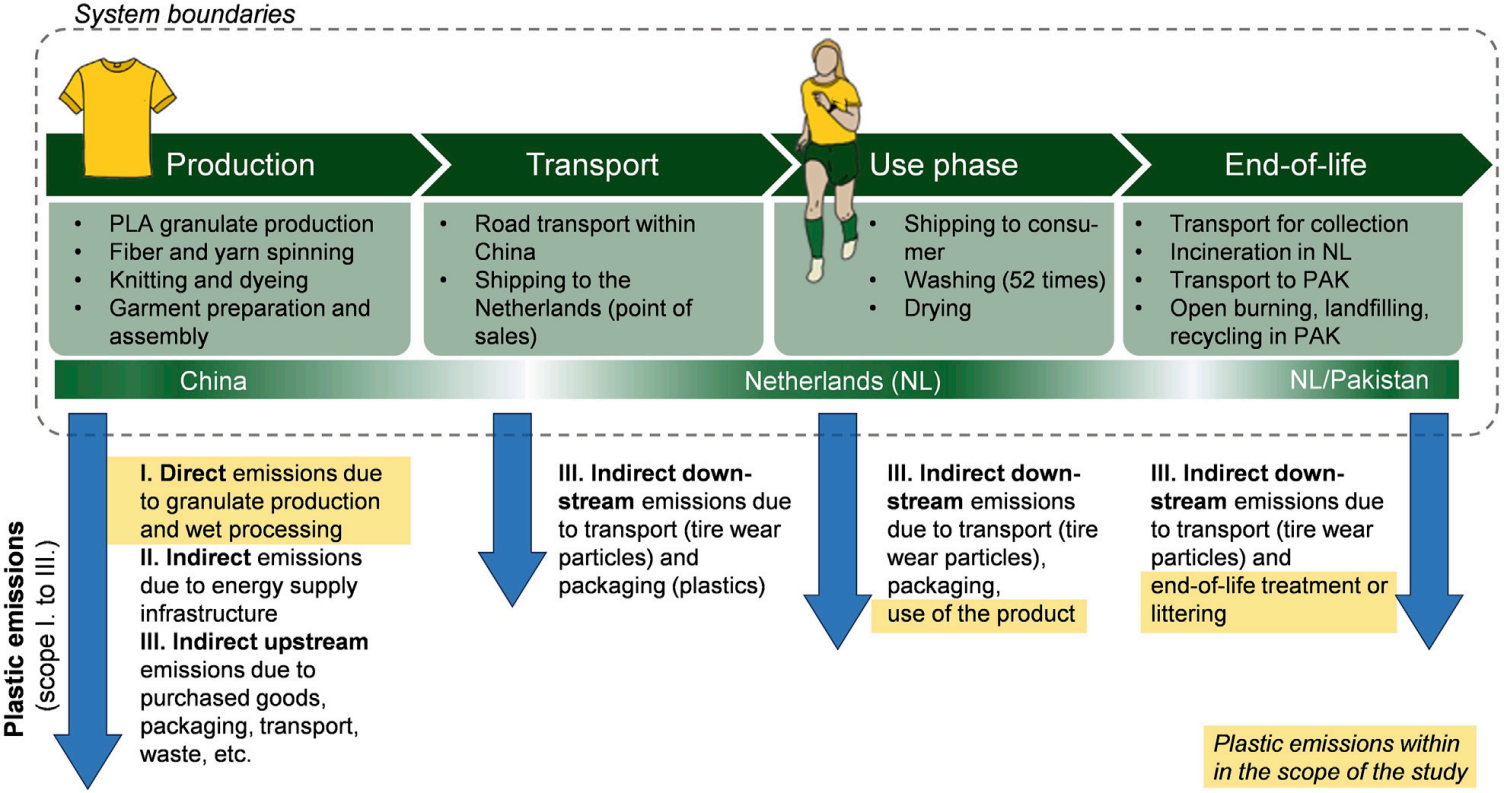
System boundaries for the LCA case study of a sports shirt. The location of the life cycle phase is given below the phase description. Plastic emissions are exemplified along the yellow arrows, differentiating between scope I., II., and III. emissions based on the greenhouse gas (GHG) protocol’s categories for emissions of GHGs (Greenhouse gas protocol 2015). Plastic emissions included within this study are highlighted in yellow.

The goal of the case study is to assess the environmental impacts of a sports shirt through an attributional LCA from cradle to grave, including the potential impacts of microplastics emissions. The functional unit (FU) was defined as “using a sports shirt weekly over a period of 1 year in the Netherlands in 2023” and included the system boundaries as shown in Figure 1. The sports shirt is assumed to consist of a monomaterial PLA without elastane or spandex which is common for loose-fit shirts (Slepian and Shiffer, 2023). Although PLA has been increasingly examined (Egan and Salmon, 2022) and used for textile applications (Mochizuki, 2022; Fattahi et al., 2020) including clothing (Sincdoo, 2025; Arapaha, 2022), datasets on sports shirts made from PLA are not yet available. Therefore we approximated the reference flow of one shirt to have a mass of 160 g as was assumed by Horn et al. (2023) for a polyester shirt.

#### 2.7.2 Life cycle inventory

Textile-related LCA literature as well as material flow analysis studies and the ecoinvent database 3.9 (Wernet et al., 2016) were used to model the life cycle inventory, covering material and energy inputs, as well as wastes and emissions associated with the life cycle. Details on each stage of the life cycle are explained in the SI (Supplementary Note S3). The included production steps and life cycle stages are depicted in Figure 1. Microparticles are released at all stages of the clothing life cycle, as wear and tear particles during production, wearing, and laundry processes and as microfibers from fragmentation of the waste clothing in landfills or the environment (Henry et al., 2019). Since there are no data available concerning the release of microparticles from PLA fiber textiles, it was assumed that polyester textile emissions are suitable to use as an approximation. Following the PLP guidelines (Peano et al., 2020) and the Plastic Footprint Network (Plastic Footprint Network, 2024), microplastic emissions from granulate production, and microfiber emissions due to wet processing steps as well as laundry and end-of-life stages were taken into account (as described in Supplementary Note S3.1). These reports have also been used in former research [e.g., by Loubet et al. (2022); Boucher et al. (2020)] and foster comparativeness among studies. Different scenarios regarding the microplastic emissions were studied as described in the scenario analysis. The plastic emission inventory is limited to direct emissions of the life cycle of the shirt, i.e., similar to scope one and partially scope three emissions (since indirect downstream emissions of the use and end-of-life phase were included; see Figure 1), comparable to the greenhouse gas protocol’s categories for emissions (Greenhouse gas protocol, 2015). Note that only emissions that end up in the marine environment were included but not to freshwater, air, or soil. Furthermore, emissions of additives and abrasion of tires due to transport were not within the scope of the study. Calculations of the plastic inventory can be found in the SI (Supplementary Note S3.1).

#### 2.7.3 Life cycle impact analysis

ReCiPe 2016 v1.03 (Huijbregts et al., 2017) was used as an impact assessment methodology, applying the Egalitarian perspective. The Product Environmental Footprint category rules served as a guidance for the modeling including allocation factors at the end-of-life (Zamporini and Pant, 2019). While the software Activity Browser (Steubing et al., 2020) was used to model the impact analysis of unit processes, calculations for the LCI and ultimate LCIA results were performed through Excel. It should be noted that this case study only serves for illustrative purposes. For the current ReCiPe 2016 methodology, EC50 (Effect Concentration 50%) data are used for toxicity impacts. However, in line with the ongoing development through GLAM, it is expected that impact assessment methodologies will adapt the toxicity assessment to EC10 data as well [as recommended by USEtox (Owsianiak et al., 2023)]. Furthermore, CO_2_ emissions resulting from the biodegradation processes (Piao et al., 2024) were not taken into account. However, the authors acknowledge that these need further examination in the future.

#### 2.7.4 Scenario analysis

Due to uncertainty regarding the end-of-life fate of exported textiles, an illustrative worst-case scenario was employed (denoted as “high” microplastic emissions in Table 4), considering that the textile waste is mismanaged and would leak into the ocean. This worst-case scenario leads to significantly higher microfiber emissions, as a fragmentation rate of 100% from macroplastics (the jersey) to microfibers was modeled [for more details on fragmentation, see Corella-Puertas et al. (2023)]. The significance of microfiber emissions related to the end-of-life of textiles has also recently been highlighted by Pinlova and Nowack (2023). As the CF depends on the size of the particle, also low (scenario 2.1, diameter of 1 µm) and high (scenario 2.2, diameter of 5,000 µm) CFs were tested to investigate the effect on the LCA results although these extreme values are not realistic. While PCL is not yet used in textile applications, the PCL CFs were applied to assess the potential effect of a more marine degradable polymer (scenarios 3). The additional rationale for this is that blending PCL with other polymers, including PLA, can have a positive effect on the biodegradation rate in seawater (Narancic et al., 2018).

**TABLE 4:** Parameters of the scenario analysis for the functional unit. BC refers to the base case scenario.

Scenario	Microplastic emissions	Characterization factor	Degradability	Rationale
BC	PLP, PFN	Medium	Low	
1	High	Medium	Low	Worst-case plastic emission scenario
2.1	High	Low	Low	Influence of small particle size and high emissions
2.2	High	High	Low	Influence of large particle size and high emissions
3.1	PLP, PNF	Medium	High	Influence of higher degradability by using the medium CF of PCL spheres and fibers
3.2	High	Low	High	Testing the lowest possible CFs of PCL, hence the greatest effect of degradability
3.3	High	High	High	Testing the highest possible CFs of PCL

Microplastic emissions are either according to the PLP and PFN guidelines for textiles, or “high” emissions considering additionally to the PLP and PFN emissions the complete fragmentation from microplastics to microfibers at the end-of-life in open dumps in Pakistan. For the characterization factors, low (1 µm) to high (5,000 µm) were used in order to test the effect of the range of CFs. Low degradability refers to PLA CFs and high degradability to PCL CFs.

## 3 Results and discussion

### 3.1 Specific surface degradation rates and discussion of effects

**FIGURE 2:**
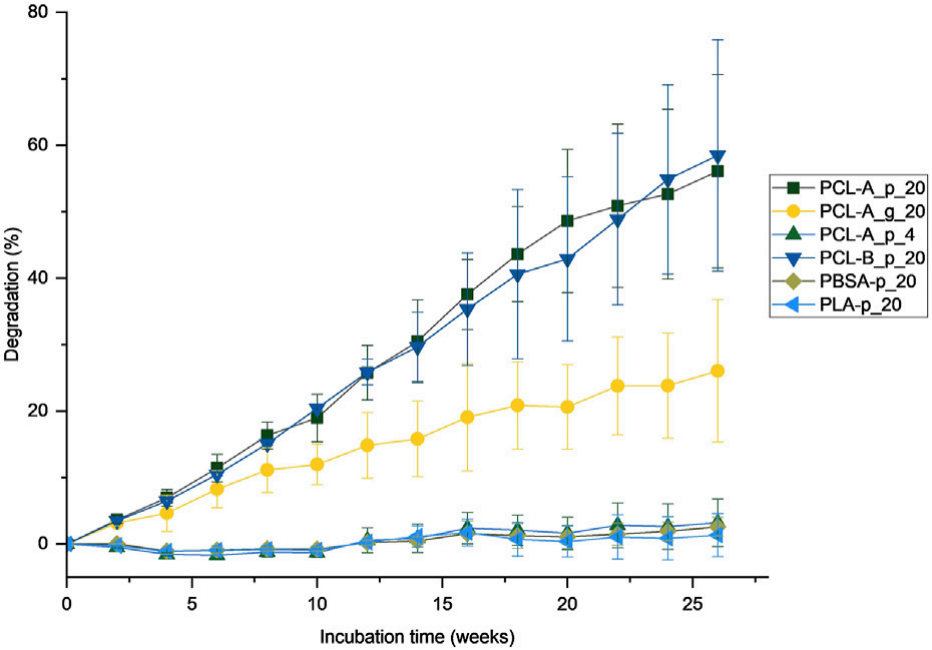
Degradation over time as the share of evolved CO_2_. The error bars indicated the standard deviation calculated based on the evolved CO_2_ for each of the three samples per triplicate. Explanation of sample handles are given in Table 2.

**FIGURE 3:**
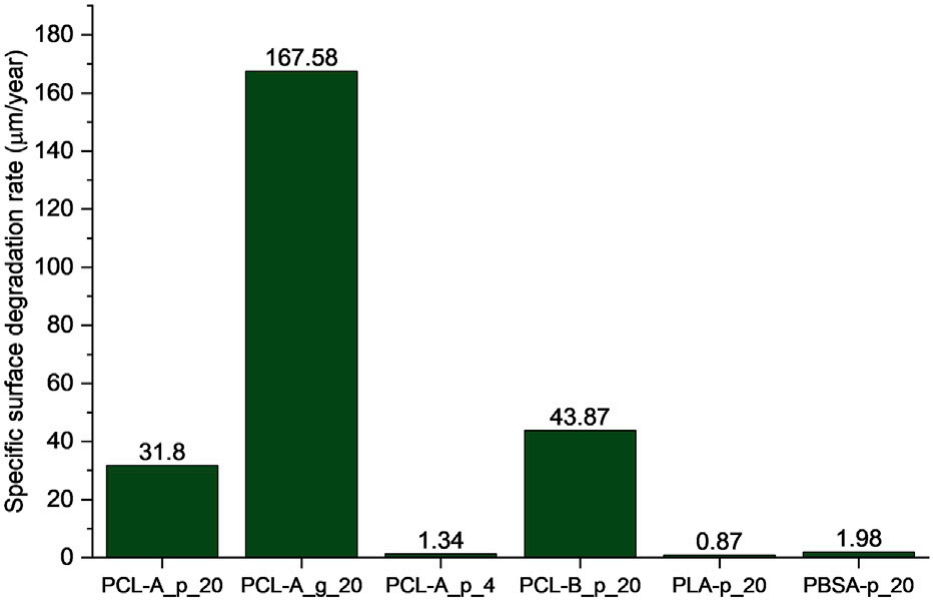
Calculated specific surface degradation rate (SSDR) based on the degree of degradation after 6 months. See Table 2 for sample details.

The degradation development is depicted in Figure 2 and an overview of the derived SSDRs is given in Figure 3. In general, the powdered PCL samples of both grade A and grade B incubated at 20°C (PCL-A_p_20 and PCL-B_p_20) have shown good biodegradation in seawater from the North Sea, which was also reported for other marine environments before (Narancic et al., 2018). After 6 months, an average degradation of 56% and 58%, respectively, was reached, showing a linear progress in degradation. Since the concentration of CO_2_ in the abiotic sample flasks remained constant, it can be assumed that the degradation of the plastics is caused by enzymes of microorganisms that metabolize the carbon present in the polymers. This was also tested by determining the cell count which showed a growth in cells for the flasks containing the polymer samples (see Supplementary Note S4). Notably, the final degradation rate within each triplicate of the powdered PCL samples at 20°C differed from 39% to 65% for PCL grade A and from 43% to 77% for PCL grade B, respectively. Due to the uncertainty range, the degradation of PCL grade A and PCL grade B did not differ significantly. Nevertheless, the observed variability is expected in biological processes which should be discussed when using these data for CF modeling. In comparison to powdered PCL of grade A, the granulate of the same grade (sample PCL-A_g_20) degraded more slowly which led to an average degradation of 26% after 6 months. Although the degradation rates of the individual samples differed as well, there was no overlap between the CO_2_-evolution of the granulate and the powdered samples. Furthermore, as enzymatic degradation is prevalent on the surface (Mohanan et al., 2020), a higher surface area of the powder leads to faster degradation (Chinaglia et al., 2018). On the other hand, abiotic degradation (e.g., through hydrolysis) can also occur in the bulk, i.e., in the inside opposed to only on the surface of the particles (Bartnikowski et al., 2019).

**FIGURE 4:**
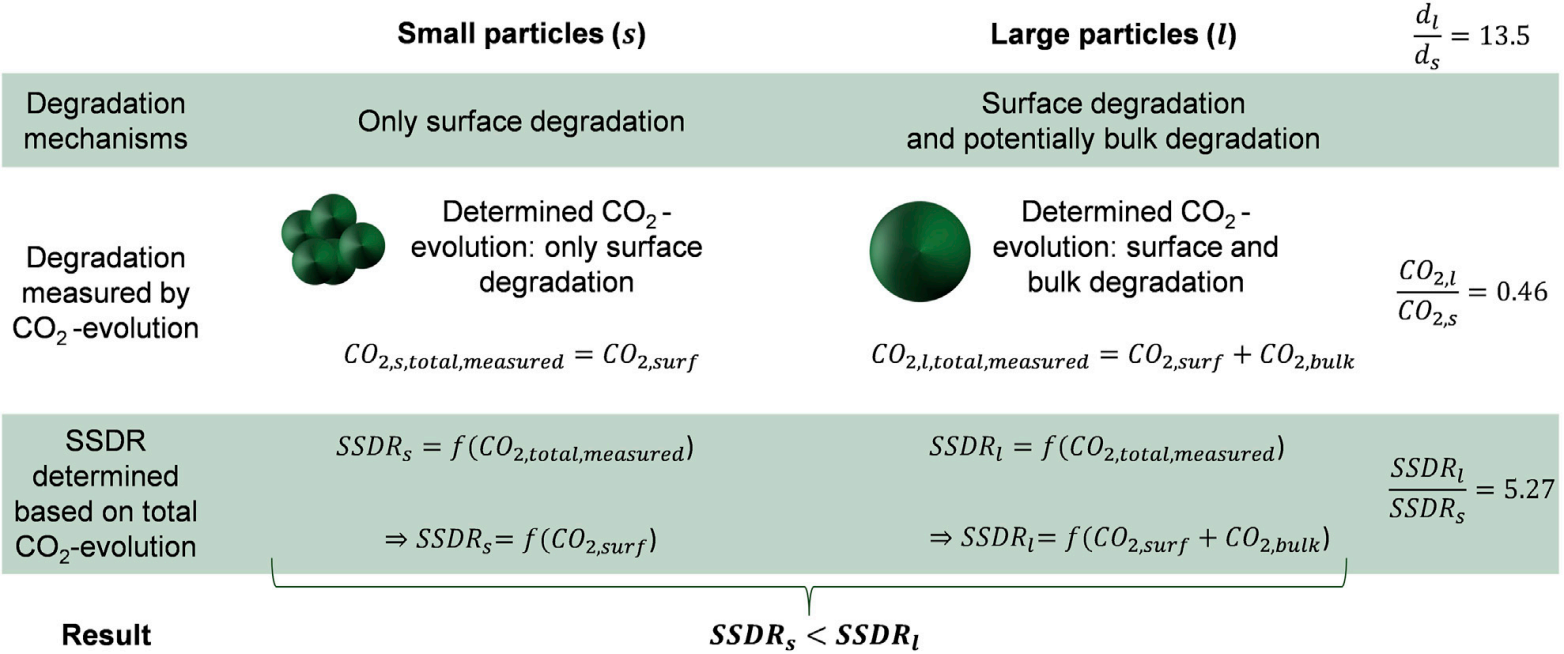
Difference in the resulting specific surface degradation rate (SSDR) comparing a smaller (s) and a larger (l) particle due to the overlap in degradation mechanisms (surface and bulk degradation). With $d_{l,s}$ being the initial diameter of the larger and smaller particle and the indices surf and bulk indicating to surface and bulk degradation, respectively.

We found different specific surface degradation rates when testing different particle sizes and only assuming surface degradation. In fact, the resulting SSDR of the granulate (sample PCL-A_g_20) is around five times higher than that of the powdered sample (PCL-A_p_20; see Figure 3). This indicates that there could be effects of bulk degradation (which depend on multiple factors such as porosity, thickness, etc.) which have also been shown for PCL elsewhere (Bartnikowski et al., 2019). In this context, we summarize through the term *bulk degradation* the abiotic degradation and biological mineralization. The abiotic degradation part, here through hydrolytic polymer chain cleavage at the ester bonds, happens in the inside of the polymer particles (Haider et al., 2019). Subsequently, depending on the porosity of the polymer matrix, the smaller oligomer chains could diffuse to the surface of the particle and be exposed to enzymatic degradation as well. The effects of those different mechanisms of surface and bulk degradation could therefore also overlap as they are not distinguishable by only assessing the CO_2_-evolution (see also Figure 4). Hence, the determined degradation of PCL granulate would be the total degradation of both surface and bulk degradation. Additionally, when using Equation 4, the influence of the initial diameter $d_{0}$ is unproportionally higher than that of the degradation term $\frac{∆m}{m_{0}}$ in the case of spheric particles. In total, the SSDR determined through the larger particles ( ${SSDR}_{l}$ in Figure 4) would hence be inaccurate when applied for a smaller particle. Therefore, when using degradation data from literature as an input for Equation 4, that are obtained for macroplastics it could lead to an overestimation of the degradation rate of smaller particles and consequently an underestimation of the physical effect on biota. A more complex degradation model would be needed to improve the SSDR determination, especially for polymers that undergo hydrolysis and bulk degradation.

When comparing the CO_2_-evolution of the samples with different initial molecular weights (samples PCL-A_p_20 and PCL-B_p_20), no significant difference was detected, as discussed before. However, a lower molecular weight would be expected to lead to faster degradation (Bartnikowski et al., 2019). The effect of differences in molecular weight is small compared to that of the initial particle size. The resulting SSDR of PCL grade B is about 38% higher than that of PCL grade A, as the mean particle size was found to be greater. This again underlines the effect of the initial diameter as was also discussed when comparing the SSDRs of granulate and powder samples.

Regarding the incubation temperature, the PCL at 4°C (sample PCL-A_p_4) showed a slower degradation evolution, leading to a total degradation of 3% after 6 months. Consequently, the SSDR of PCL at 4°C (1.98 μm/year) is around 96% lower than the SSDR at 20°C. Whilst most degradation data are published for elevated temperatures, actual degradation in the natural aquatic environment proceeds more slowly as the actual temperature in the oceans is usually lower [depending on the depth and location (Copernicus Programme, 2024)]. This aspect is particularly relevant for non-buoyant plastics emissions as the particles will sink to the ground of the maritime compartment where the temperatures are lower than at the surface. When using the degradation rates obtained at higher temperature, the emission’s residence time in the marine compartment depending on the degradation rate would be underestimated. Comparing the SSDRs to values in literature (Maga et al., 2022), the obtained SSDRs of this work were mostly smaller (up to 486 times; see Table 5). The values are closest when considering the SSDR derived from PCL granulate samples which have a similar range of size as the samples assessed in the literature. However, it should be noted that the experimental conditions were different in terms of environment, temperature, and degradation determination.

**TABLE 5:** Comparison of obtained SSDRs of PCL samples to values in Maga et al. (2022).

Literature SSDR	Sample	Conditions	Ratio of literature SSDR to PCL-			
			A_p_20	A_g_20	A_p_4	B_p_20
260.71 (Heimowska et al., 2017)	Film, 0.06 mm thickness	Weight loss; natural conditions at 17.6°C–20.3°C); Baltic Sea water	8.2	1.6	194.3	5.9
365.00 (Kasuya et al., 1998)	Film, 10 mg, 0.1 mm thickness	BOD biod., 25°C, seawater Pacific Ocean	11.5	2.2	272.1	8.3
514.91 (Kasuya et al., 1998)	Film, 10 mg, 0.1 mm thickness	BOD biod., 25°C, seawater (bay, Tokyo)	16.2	3.1	383.8	11.7
436.70 (Kasuya et al., 1998)	Film, 10 mg, 0.1 mm thickness	Weight loss biod., 25 °C, seawater Pacific Ocean	13.7	2.6	325.5	10.0
651.79 (Kasuya et al., 1998)	Film, 10 mg, 0.1 mm thickness	Weight loss biod., 25°C, sea water (bay, Tokyo)	20.5	3.9	485.8	14.9
91.25 (Lepoudre, 2017)	Film, 0.1 mm thickness	-, 27°C, seawater	2.9	0.5	68.0	2.1

The factor shows the ratio between the value from Maga et al. (2022) and the obtained values for PCL samples. A factor greater than 1 indicated that the SSDR of Maga et al. (2022) is higher. See Table 2 for sample details.

For PLA, around 1% of degradation was observed after 6 months of incubation. This leads to an SSDR of 0.87 μm/year. This finding is in line with data in the literature, where PLA was found to be degradable only in industrial composting. However, the degradation under home composting conditions [e.g., as shown by Mercier for an incubation time of 450 days (Mercier et al., 2017)] or in seawater is negligible. Recently, it has also been reported by Royer et al. (2023) that PLA fibers did not show any biodegradation in seawater after >400 days of incubation. Gerritse et al. (2020) determined a fragmentation rate of around 7% for PLA in seawater but biodegradation was not assessed. Therefore, the obtained results are coherent with former research (Narancic et al., 2018). Maga et al. (2022) proposed an adjusted SSDR of 0.001 μm/year based on Lepoudre (2017) for PLA in marine water, suggesting that there is no biodegradation evident for PLA.

PBSA showed an average degradation of 3%, resulting in an SSDR of 1.98 μm/year which is 120% higher than the SSDR of PLA. Compared to the SSDRs proposed by previous research (Maga et al., 2022), the obtained value is significantly lower, as those range from 26.07 to 221.61 μm/year. These deviations could be caused by the differences in the inoculum (seawater from Tokyo Bay vs. Oostende) and the type of degradation assessment (mass loss vs. CO_2_ measurements).

Ultimately, it should be noted that the calculated degradation evolution will deviate from the actual one as also residual monomer content affects the interpretation (Klaeger et al., 2019). This needs to be included in further studies. However, by analyzing the CO_2_-evolution instead of directly measuring mass loss, fragmentation can be excluded as a potential source of uncertainty which in turn increases the accuracy of the obtained SSDRs.

### 3.2 Resulting CFs and comparison

Based on the derived SSDRs of the tested polymers, the characterization factors were developed for microspheres, microfibers, and microfragments of films considering five initial diameters or film thicknesses from 1 to 5,000 µm. The shapes and sizes were chosen in order to be consistent with the existing MarILCA CFs for other polymers (Corella-Puertas et al., 2023 ^1^). The proposed values for both midpoint and endpoint categories are provided in Table 6. As the same EEFs (for seawater and sediments, following Saadi et al.^1^) apply for all polymers, the differences in CFs are directly linked to differences in the FFs (see Equation 7) as CFs solely combine FFs and EEFs. Ultimately, the fate factor is a function of the SSDR, the particle size and shape as well as the sedimentation, resuspension, and deep burial rates. As this work focuses on the degradation rate, the effects of the corresponding components (SSDR, particle size, particle shape) will be discussed below.

$$\begin{array}{c} \text{CF}\propto \text{FF},\\ \text{FF}=f\left(\left\{\begin{array}{c} Degradation\;rate=f\left(\left\{\begin{array}{c} SSDR\\ Particle\;surface=f\left(\left\{\begin{array}{c} Particle\;size\\ Particle\;shape\; \end{array}\right.\right) \end{array}\right.\right)\\ Sedimentation\;rate\\ Resuspension\;rate\\ Deep\;burial\;rate \end{array}\right.\right) \end{array}$$ (7)

**TABLE 6:** Midpoint and endpoint characterization factors proposed for physical effects on biota of microplastics from PCL (based on the geometric average of the obtained SSDRs for PCL), PLA, and PBSA.

Polymer	Shape	Size (µm)	Midpoint CF (PAF*m^3^*day/kg_emitted_)	Endpoint CF (PDF*m^2^*year/kg_emitted_)
PCL	Sphere	5,000	5.01E+07	1.36E+03
		1,000	1.34E+07	3.64E+02
		100	1.38E+06	3.77E+01
		10	9.55E+04	2.60E+00
		1	3.24E+03	8.83E- 02
	Fiber	5,000	6.09E+07	1.66E+03
		1,000	1.73E+07	4.72E+02
		100	1.87E+06	5.08E+01
		10	1.39E+05	3.77E+00
		1	5.08E+03	1.38E-01
	Film	5,000	7.80E+07	2.12E+03
		1,000	2.46E + 07	6.70E+02
		100	2.83E+06	7.69E+01
		10	2.29E+05	6.23E+00
		1	9.50E+03	2.58E-01
PLA	Sphere	5,000	1.84E+08	5.01E+03
		1,000	1.25E+08	3.39E+03
		100	2.87E+07	7.81E+02
		10	3.29E+06	8.94E+01
		1	2.79E+05	7.59E+00
	Fiber	5,000	1.90E+08	5.17E+03
		1,000	1.38E+08	3.76E+03
		100	3.64E+07	9.91E+02
		10	4.38E+06	1.19E+02
		1	3.89E+05	1.06E+01
	Film	5,000	1.97E+08	5.35E+03
		1,000	1.56E+08	4.23E+03
		100	4.98E+07	1.36E+03
		10	6.53E+06	1.78E+02
		1	6.13E+05	1.67E+01
PBSA	Sphere	5,000	1.56E+08	4.25E+03
		1,000	8.10E+07	2.20E+03
		100	1.31E+07	3.57E+02
		10	1.34E+06	3.65E+01
		1	9.44E+04	2.57E+00
	Fiber	5,000	1.66E+08	4.53E+03
		1,000	9.50E+07	2.58E+03
		100	1.71E+07	4.65E + 02
		10	1.81E+06	4.92E+01
		1	1.37E+05	3.72E + 00
	Film	5,000	1.78E+08	4.85E+03
		1,000	1.15E+08	3.13E+03
		100	2.45E+07	6.67E+02
		10	2.73E+06	7.44E+01
		1	2.25E+05	6.13E+00

Additionally, midpoint and endpoint characterization factors show similar trends due to constant conversion factors. Therefore, the following discussion will be focused on midpoint characterization factors but is similarly applicable to the developed fate factors and endpoint CFs. This work is differentiating from previous research by Corella-Puertas et al. (2023) as also the fate in the sediment compartment was included which was based on Saadi et al.^1^. It should be noted though that the same degradation rate was assumed for the sediments as in the water column [following the approach by Saadi et al.^1^] although only degradation in seawater was experimentally determined in this work. This is due to the lack of sediments specific degradation data (Maga et al., 2022).

**FIGURE 5:**
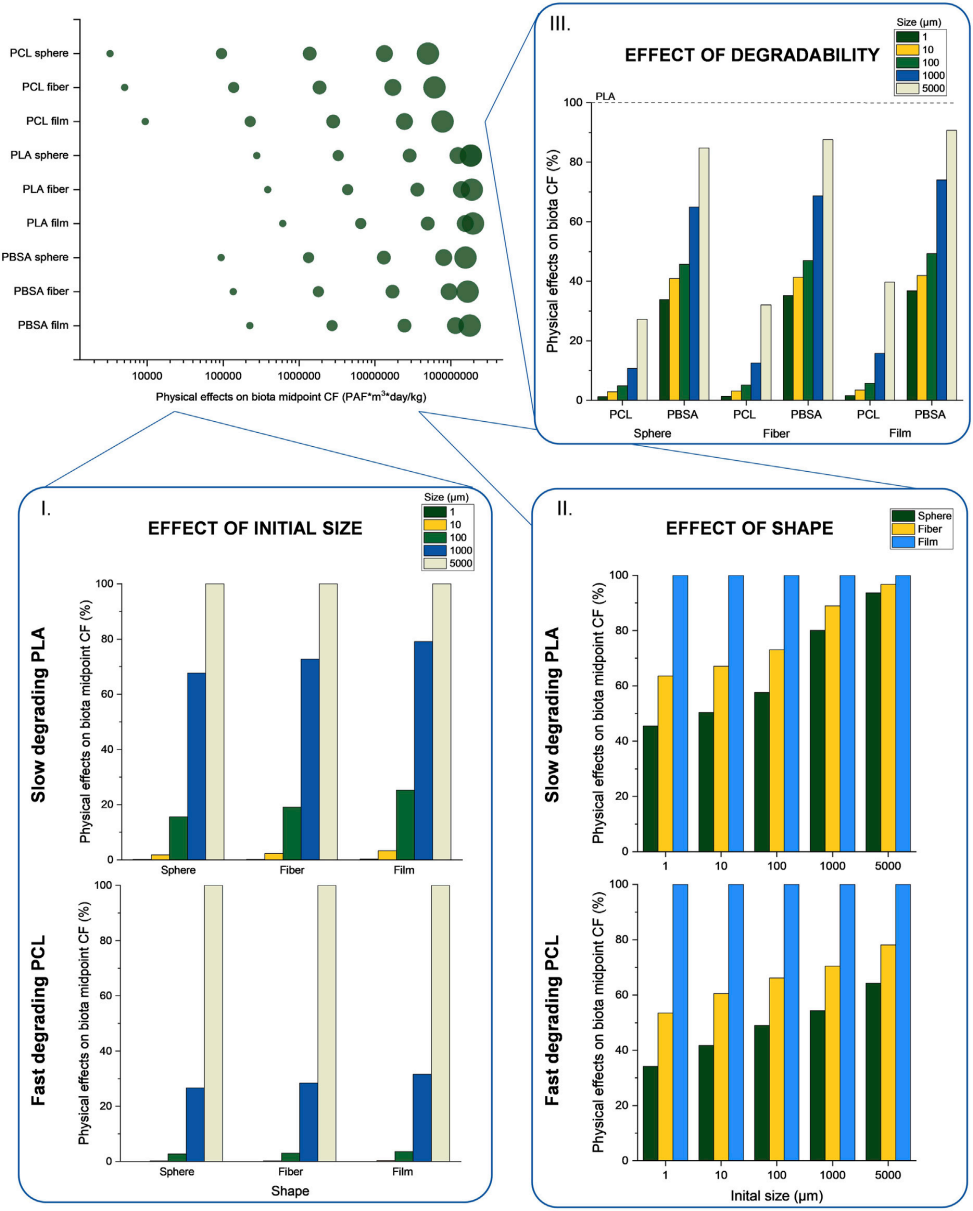
Resulting physical effects on biota characterization factor (CF) (the size of the circle indicates the diameter or thickness of the particles, from smallest to largest 1, 10, 100, 1,000, and 5,000 µm) with a focus on I. the effect of size on the CF compared for each shape, II. the effect of shape on the CF compared for each initial size, and III. the effect of degradability (showing the CFs of PCL and PBSA relative to the according CF of PLA which represents 100%). Every size is given in µm.

The resulting CFs are shown in Figure 5 and isolated effects of initial size, shape, and degradability are highlighted by showing relative CFs (in I. to III.). As PLA showed the lowest SSDR, it serves as an example of a very slowly degrading polymer, in contrast to PCL which is an example of a highly degradable polymer. Therefore, the discussion will be focused on PLA and PCL to identify the effects of differences due to degradability. Since the density of the studied polymers is greater than 1.1 g/cm^3^, they are considered to belong to the high-density polymer group (Corella-Puertas et al., 2023). Consequently, only effects pertaining to that group are discussed while effects in middle- and low-density polymer groups can be found in Corella-Puertas et al. (2023). In general, the proposed midpoint CFs for PCL, PLA, and PBSA range from 3.24E+03 to 1.97E+08 PAF*m^3^*day/kg_emitted_ (see Figure 5). As mentioned above, the fate of the polymer particle in water and sediments, and hence its FFs and CFs are a function of the removal and transfer mechanisms of degradation, sedimentation, resuspension, and deep burial. The degradation depends on the polymer itself (through the SSDR), and the shape and size of the particle (through the surface and therefore degradable area relative to the volume of the particle). The sedimentation rate however only depends on the density of the polymer, following the current methodology by Corella-Puertas et al. (2023). Further, the resuspension and deep burial rates are independent of the density, size, shape, or polymer (Quik et al., 2023). For high-density polymers, the residence time in the marine water compartment is short, and therefore the transfer mechanism of sedimentation is more influential than the one of degradation, as particles do not have time to degrade before reaching the sediments. Saadi et al.^1^ have shown that a large fate in water or sediments were both drivers of high CFs. In fact, exposure and effect is higher in the water column (EEF_w_) leading to high CFs for large, low and medium density polymers which have long fate in water (Saadi et al.^1^, Corella-Puertas et al., 2023). On the other hand, exposure and effect is smaller in the sediments (EEF_sed_), but the dilution volume is much smaller (Hajjar et al., 2024), making concentrations of microplastics significantly higher in sediments that in water, for a similar fate. This means that a large fate in sediments, for large high-density polymers, is also a driver of high CFs as their model considers the volume of the compartment in which the particles are Saadi et al.^1^). The most significant effect was identified for spheric particles of the fast-degrading PCL where the difference between a 1 µm and a 5,000 µm particle accounts for 99.99%. On the other hand, other transfer and loss rates (i.e., resuspension and deep burial) are of smaller importance. In fact, these two rates, which are constant for all polymers, sizes, and shapes), are two to four orders of magnitude smaller than degradation and sedimentation (Quik et al., 2023; Corella-Puertas et al., 2023 ^1^). Therefore, once polymer particles reach the sediments their fate is largely dictated by the degradation that will occur in this compartment, and only a small amount will resuspend within the water column or reach a deeper layer of the sediments via deep burial. However, the bigger the particle is when it reaches the sediments, the more likely it is to resuspend as large particles have a longer fate in the sediments (up to 100 years for large PLA films, see Supplementary Note S1). This will however have a small effect on the overall CF as the resuspension rate is much smaller than the sedimentation rate.

When comparing the CFs of differently shaped particles at the same initial size (see Figure 5II), it is shown that the CFs of film particles are the highest (i.e., the CFs are displayed compared to the film CF of each particle size in Figure 4 II.; note that a relative scale was chosen rather than an absolute scale to isolate the effect of shape). This phenomenon is due to the small surface-to-volume ratio and hence lower degradation rate. Regarding the PLA CF for large particles, the shape of the emission also has a small influence (e.g., 6% for spherical particles with an initial diameter of 5,000 µm), as the rate of sedimentation dominates the fate of the particles. The most significant effect of the shape for PLA is found for particles of 1 µm where the CF of a spheric particle is 54% smaller than that of a film particle. For a fast-degrading polymer such as PCL the shape of the emission has a more significant influence on the resulting CFs than for PLA, regardless of the initial particle size. This different CFs trend between fast and slow degrading particles (see Figure 5II) can also be explained by the different compartmental impacts between each particle. In fact, slow degrading PLA particles have time to reach the sediments, and therefore all particles size have a large fate and potential impacts in this compartment. On the other hand, fast degrading PCL has larger impact in the water compartment for particles of 1 and 10 μm, while the impact is larger in sediments than in water for 100, 1,000 and 5,000 µm particles. Comparing a fiber to a film particle of the same initial size has an influence of 47% or more. However, in total, the shape of the emission results in smaller differences, within the same order of magnitude, in the characterization factor than the size of the particle.

In order to analyze the effects of the differences in SSDRs, Figure 5III. shows the CFs of PCL and PBSA relative to PLA, expressed as the proportion of the CFs of PCL and PBSA particles of the same diameter and shape, with PLA set as the 100% reference (e.g., the CF of a 10 µm PCL sphere is set in proportion to the CF of a 10 µm PLA sphere). This aspect is especially interesting when considering higher degradable polymers for specific applications and modeling their impact through LCA. In this work, regardless of the emission shape, the faster degrading PCL leads to a significant reduction in the CF compared to the one of PLA, by up to 99%. Next to that, also the CFs of PBSA showed a decrease in CF compared to PLA of up to 63%. As previously discussed, it is observed that the CFs of larger particles are less influenced by a higher SSDR than smaller particles.

Comparing the influence of the initial size, the shape, and the degradability shows that the initial size can have the highest influence on the resulting CF while the shape of the emission affects the CFs the least. Therefore, special emphasis should be placed to correctly determining the size of the plastic emission when using the CFs in an LCA study. However, the current CF modeling only takes into account the fate-mechanisms of degradation, sedimentation [as initially developed by (Corella-Puertas et al., 2023; Corella-Puertas et al., 2022)], resuspension, and deep burial [as proposed by Saadi et al.^1^] since these have been considered to be the most dominant phenomena especially for non-buoyant polymers that accumulate in the sediments. Additional mechanisms such as windage, Langmuir cells, biofouling effect, etc. (Hajjar et al., 2024) need to be included in further developments of the model.

### 3.3 Comparison of CFs to literature

**FIGURE 6:**
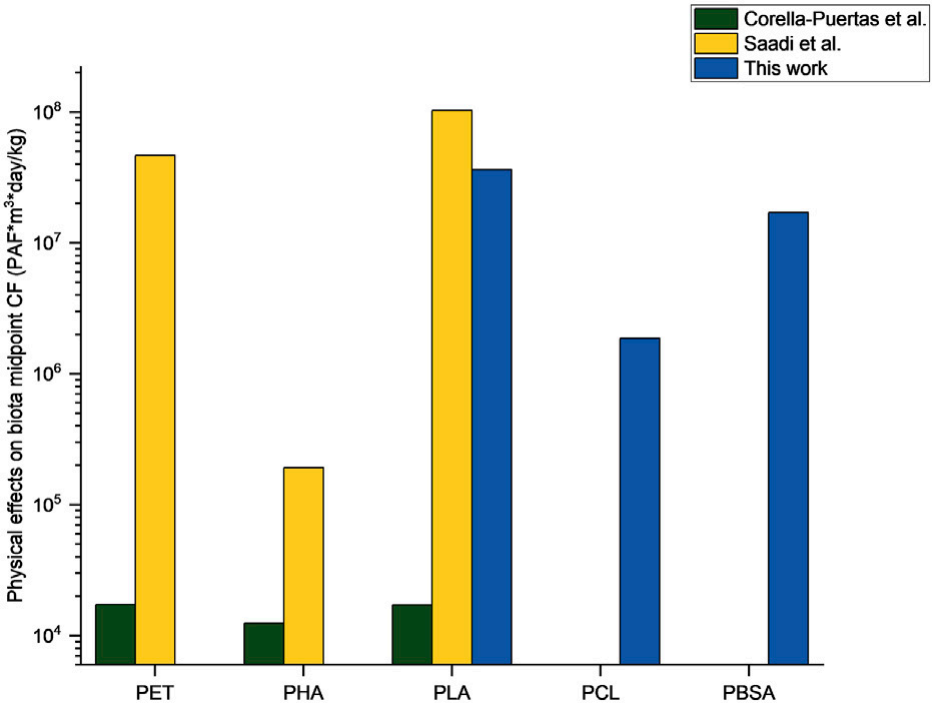
Comparison of developed characterization factors to CFs of previous research by Corella-Puertas et al. (2023) and Saadi et al.^1^ (considering values for polymer fibers at an initial diameter of 100 µm).

Marine microplastic CFs have also been published by Maga et al. (2022) and recently by Schwarz et al. (2024). However, a quantitative comparison to those CFs is not possible due to differences in the modeling approaches (e.g., regarding the time horizon, compartments included, and the fate model). As this work is a continuation of (Corella-Puertas et al., 2023) and Saadi et al.^1^, the quantitative comparison will be limited to the aforementioned studies. The resulting comparison is shown in Figure 6, considering the CFs for an initial fiber diameter of 100 µm which is the same order of magnitude as the characteristic length determined for the incubated polymer samples. The CFs of Corella-Puertas et al. (2023) did not include the fate in sediments yet. Based on Corella-Puertas et al. (2023), Saadi et al.^1^ developed an approach to also integrate the sediment compartment. In general, the CFs developed in this work are in line with the observation found in Corella-Puertas et al. (2023) and Saadi et al.^1^ where it was shown that the CFs of high-density polymers are the lowest. However, these results show that the size of high-density particles has a large influence on the CFs, which was not the case in the previously computed CFs (Corella-Puertas et al., 2023). The authors had in fact shown that size was an important factor for low- and medium-density polymers only. The CFs of PCL, PBSA, and PLA are up to three order of magnitude higher compared to other non-buoyant polymers such as PET in Corella-Puertas et al. (2023). These differences are due to the addition of fate, exposure, and effect in the sediments, where larger particles will have a larger fate, and thereby higher impacts, which were not considered in Corella-Puertas et al. (2023). Further, in this compartment, a higher fraction of biota is potentially affected due to the smaller volume and thus a higher species concentration. When directly comparing the PLA CFs, the CF of this work is three orders of magnitude higher than the CF published by Corella-Puertas et al. (2023). Nevertheless, the SSDR defined in this work is within the PLA SSDR uncertainty range determined by Corella-Puertas et al. (2023) which validates the former work. Compared to the PLA CF of Saadi et al.^1^, the PLA CF of this work is one order of magnitude lower. The difference is caused by a higher SSDR and therefore faster degradation. Therefore, the developed CFs can be seen as an update to the CFs proposed by Corella-Puertas et al. (2023) ^1^.

### 3.4 Case study: microplastic influence on ecosystem quality

**FIGURE 7:**
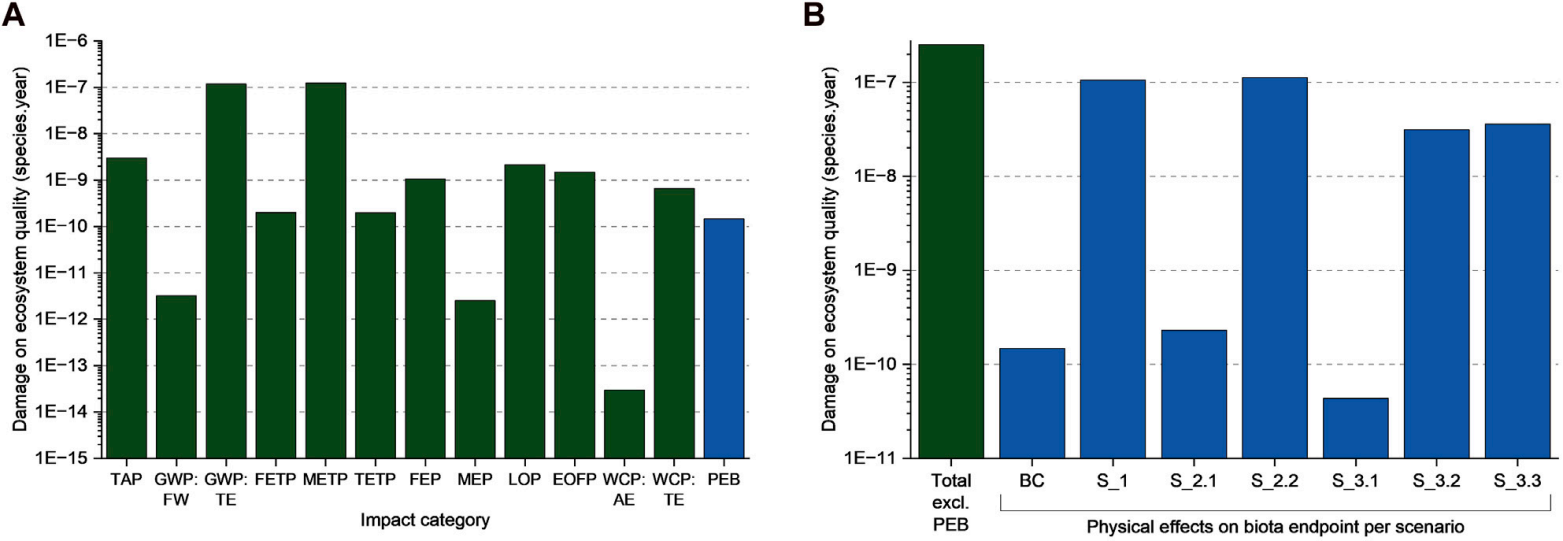
Damage to ecosystem quality caused by the functional unit of the LCA case study. **(A)**. Impacts per category of the base case plastic emissions (TAP, terrestrial acidification potential; GWP: FW, global warming potential impact on freshwater; GWP: TE, GWP impact on terrestrial ecosystems; FETP, freshwater ecotoxicity potential; METP, marine ecotoxicity potential; TETP, terrestrial ecotoxicity potential; FEP, freshwater eutrophication potential; MEP, marine eutrophication potential; LOP, agricultural land occupation potential; EOFP, Photochemical oxidant formation: terrestrial ecosystems; WCP: AE Water use: aquatic ecosystems; WCP: TE, Water use: terrestrial ecosystems; PEB, physical effects on biota). **(B)**. Resulting physical effects on biota impact of the base case (BC) and the scenarios S_1 to S_3.3, compared to the sum of damage due to the other impact categories. See Table 4 for details on scenario descriptions.

The CFs developed in this work were applied to a textile case study of a running shirt. Detailed results for individual impact categories and life cycle stages of the case study can be found in the SI in Supplementary Note S5. In the following section, the discussion will be focused on the endpoint category *damage on ecosystem quality* as this is the area of protection that would be affected by physical effects on biota caused by marine microplastic emissions. As shown in Figure 7A, the damage to ecosystem quality due to physical effects on biota is very small compared to damage originating from the global warming and marine ecotoxicity potential (by three orders of magnitude). In the base case scenario (see Section 2.7.1), microplastics would account for a share of 0.06% of the total endpoint category. This contribution is in the same order of magnitude as the share of the freshwater and terrestrial ecotoxicity potential and greater than the share of the eutrophication potential. However, it should be noted that for the present work only microplastic emissions to the ocean were considered (thus emissions to freshwater, air, and soil were not included), and toxic effects of plastic additives and degradation products are also not within the scope of the impact category.

The scenario analysis allowed to study different assumptions and effects. In scenario (1), the worst-case microplastic emissions were assumed because of mismanaged waste textiles. This would lead to an increase in physical effects on biota by three orders of magnitude. Considering the smallest particle size of the emission compared to the largest particle size, the difference in impact changes by three orders of magnitude. Comparing the results of scenario (1) and (2.1) shows that considering a small compared to a medium particle size results in an impact reduction of more than 99%. These observations point out that the impact of microplastic emissions is more dependent on the inventory than on the applied characterization factor. This is also evident when considering the CFs of PCL as a highly degrading polymer [scenario (3)]. Compared to base case, the impact of microplastic emissions is reduced by 71% and therefore one order of magnitude. If the entire shirt would be lost to the environment and a fragmentation rate of 100% was assumed, physical effects on biota would account for up to 31% of total damage on ecosystem quality of the functional unit. In that case, microplastics belong to the main drivers of damage to ecosystem quality as also the global warming potential of the production and use phase. If electrification and green energy would lead to a hypothetical reduction of 50% of global warming impacts, then the relevance of physical effects on biota could increase, as the contribution to the endpoint category would rise by 31%. Nevertheless, in the base case the contribution and therefore relevance remains small when compared to the global warming and ecotoxicity potentials. In general, it should be noted though that the impact does not depend on the locations of the emission (although in the present case these would occur in China, the Netherlands, and Pakistan) as the CFs are not yet regionalized.

Switching from a non- or slowly degrading polymer (in this case PLA) to a highly degrading alternative (PCL) could lead to a microplastic impact reduction in the marine environment of 71% which is indicated by comparing the base case to scenario (3.1) in Figure 7B. Although this reduction is significant when only considering physical effects on biota, it only leads to a very small improvement of the overall damage to ecosystem quality which is due to the small contribution of microplastics to the endpoint category. It should be noted though that also the impacts of the conventional impact categories would change if PCL would be used instead of PLA. However, these results are only preliminary and need to be examined in more depth including issues such as toxicity of additives and degradation products, and emissions to other environmental compartments. Thus, based on this work, recommendations for or against the use of biodegradable fibers in clothing applications cannot be given. Indeed, the environmental consequences need further case-by-case examination, evaluating biobased and biodegradable polymer alternatives for particular products rather than generalized decision-making. Other aspects that are correlating with biodegradability properties (e.g., insufficient mechanical properties of polymers that are more suitable for enzymatic degradation) could counterbalance the positive effect of minimizing the marine microplastic impacts.

### 3.5 Correction factor for SSDR determination

This study aimed to validate the modeling approach of CFs based on literature for degradation data [as it was done, e.g., by Maga et al. (2022); Corella-Puertas et al. (2023)]. It was demonstrated that using macroplastic degradation data to approximate microplastic surface degradation can lead to distortions and overestimations of the degradation rate (see Figure 4 in Section 3.1). To prevent this, we propose the use of a conservative correction factor if only CO_2_-evolution based macroplastic degradation data is available. For adjusting the mass loss based on CO_2_-evolution of a polymer from a greater scale to a smaller scale, the following factor $f_{corr}$ is proposed (see Equations 8, 9):

$$f_{corr}={\left(\frac{d_{s}}{d_{l}}\right)}^{\frac{2}{3}}$$ (8)

$$\frac{∆m}{{m_{0}}_{corr}}=f_{corr}\times \frac{∆m}{{m_{0}}_{l}}$$ (9)

With $d_{s}$ and $d_{l}$ being the initial diameters of the smaller and larger particles and $\frac{∆m}{{m_{0}}_{l}}$ the mass loss over the degradation time of the larger particle. To determine the SSDR of the microplastic (i.e., smaller particle), which is necessary for the FF, the corrected mass loss is used in Equation 4. The altered mass loss does not represent a physically correct figure but serves as a conservative approximation of the actual specific surface degradation rate that would be present for a smaller microplastic particle. However, the authors want to point out that the preliminary correction factor only applies to this material (the tested PCL) and needs to be tested for other polymers, particle sizes, and shapes (see Supplementary Note S6 for explanation).

## 4 Conclusion and outlook

Through biodegradation experiments, datasets for the determination of specific surface degradation rates of three biodegradable polymers were derived. The comparison between different experimental and sample conditions (aligned with Goal 1 of this study) revealed that the size of the tested polymer particle as well as the temperature have a large effect on the resulting SSDR. Contrarily, the grade of the plastic (in this case PCL) did not have a significant influence on the SSDR. Furthermore, this work proposes an updated set of characterization factors for these biodegradable polymers that allows to refine the modeling of their impact in marine ecosystems, considering three different shapes and five different sizes (Goal 2 of this study). As such, it builds up on the work by Corella-Puertas et al. (2023), Corella-Puertas et al. (2022), and Saadi et al.^1^. Studying the parameters of the calculation model (initial size, shape, and degradability) showed that the initial size of the emission can have the highest influence on the resulting CF while the shape of the emission affects the CFs the least. According to Goal 3, the developed CFs were tested in a case study of a sports shirt, revealing that the damage on ecosystem quality caused by physical effects on biota is smaller than the damage caused by other impact categories, especially climate change. However, the mismanagement of textile waste and its emission and fragmentation in the marine environment can lead to a magnitude of damage on ecosystem quality that is comparable to damage caused by the global warming potential of the case study. Furthermore, it was shown that switching to marine biodegradable plastics for textile fibers could lead to a microplastic impact reduction in the marine environment of up to 71%. However, as the damage on ecosystem quality caused by climate change and other impact categories is much higher, the potential of biodegradable fibers needs to be assessed more holistically.

### 4.1 Recommendations for future research on experimental and LCA side

This work proposes steady state fate and therefore characterization factors, in contrast to Maga et al. (2022) and Schwarz et al. (2024) where FF for different time horizons (individual, hierarchist, egalitarian) are presented. Further time horizons could therefore be developed in future research.

The documentation for the experiments can serve as an example for future research collaboration in this field to improve the applicability of datasets to CF modeling. Furthermore, the proposed CFs are only applicable for physical effects on biota. However, it was shown that degradation products of PCL can have toxic effects on aquatic biota (Tamayo-Belda et al., 2022). These effects need to be considered to foster a holistic assessment. Next to that, additives released have been omitted in this study but need to be addressed in future research. Research to integrate this aspect into LCA is currently ongoing within the MarILCA group. Another limitation of the present study is the lack of degradation rates in marine sediments, as the same degradation rates for both the water column and the sediments are assumed. Hence, more experimental research is imperative to evaluate the degradation rate in sediments, especially for non-buoyant polymers. Furthermore, the possibility of bulk degradation needs to be examined through additional experimental analysis. The proposed characterization factors could additionally contribute to a regionalized assessment as the data are particularly applicable to the North Sea water. Recommendations to facilitate the dataflow between experimental research and CF modeling are summarized in Table 7. Ultimately, it should be pointed out that closer collaboration between experimental and LCA research is an integral part of improving the environmental assessment of plastics and their alternatives (Askham et al., 2023).

**TABLE 7:** Recommendations for future experimental research to determine characterization factors of microplastics in LCA.

Necessity	Parameter	Explanation
1	Size and shape	Determination of size distribution and shape of the microparticles prior to incubation, e.g., through microscopic imaging; for calculation of the surface area of the particles
1	Polymer density range	Determining the sinking behavior of the polymer in sea (and fresh)water
1	CO_2_ evolution or O_2_ consumption over time + blank and abiotic samples	Necessary to evaluate the degradation rate over time; mass loss is not sufficient due to fragmentation processes Blank samples with only seawater (to assess baseline CO_2_ evolution) and abiotic samples (to eliminate other sources of CO_2_ production aside from bacterial activity)
1	Polymer total carbon content (C_tot_)	Necessary to assess the degree of biodegradation based on the total carbon present in the sample
1	Sample mass/concentration	Necessary to assess the total carbon source due to the added polymer
2	Temperature	Testing at two different temperatures (e.g., 20°C and 4°C) to determine the difference between surface water and deep water degradation
2	Exact polymer density	Reporting of the exact polymer density can improve fate modeling of polymer particles
2	Location of seawater sample	Reporting the location of the sampled seawater can support future developments in regionalized characterization factors
3	Cell count	Monitoring the cell count ensures the positive evolution of microorganisms
3	Additives	If possible, reporting known additives can leverage further developments in incorporating toxicity
3	Degradation products	If possible, disclosing the reactivity and toxicity of known degradation products (such as monomers) can leverage further developments in incorporating toxicity

Necessity listed from 1 (high) to low (3). Necessity 1 indicates that these data are relevant to compute the characterization factors using the current methodology (Saadi et al.^1^, Corella-Puertas et al., 2023). Necessity 2 data would refine the current model and aid the efforts to regionalize the CFs. Low necessity (3) is given to parameters that could be useful in developments of characterization factors that also incorporate toxicological effects on biota.

## Data Availability

The original contributions presented in the study are included in the article/Supplementary Material, further inquiries can be directed to the corresponding author.

## Footnotes

1 Saadi, N., Lavoie, J., Fantke, P., Redondo-Hasselerharm, P., Boulay, A.-M. (2025). Including impacts of microplastics in marine water and sediments in life cycle assessment. *J. Clean. Prod.* (under review).

## Abbreviations

bioPET, biobased polyethylene terephthalate; CF, characterization factor; EEF, exposure and effect factor; EEF_sed_, EEF for sediments; EEF_w_, EEF for water column and surface water; FF, fate factor; FU, functional unit; LCA, Life Cycle Assessment; LCI, life cycle inventory; LCIA, life cycle impact assessment; MarILCA, Marine Impacts in Life Cycle Assessment; PAF, Potentially Affected Fraction of species; PBSA, poly (butylene succinate); PDF, Potentially Disappeared Fraction of species; PCL, poly (ε-caprolactam); PFN, Plastic Footprint Network; PLA, poly (lactic acid); PLP, Plastic Leak Project; POM, Polarized Optical Microscope; SI, supplementary information; SSDR, specific surface degradation rate.
