# Supplementary material for: Modeling marine microplastic emissions in Life Cycle Assessment: characterization factors for biodegradable polymers and their application in a textile case study
Source: Front Toxicol. 2025 Mar 17;7:1494220. doi: 10.3389/ftox.2025.1494220 (PMC11962433; doi:10.3389/ftox.2025.1494220)
Supplement: Supplementary file 1 [file Supplementaryfile1.docx]

**Supplementary Information for:**

**Modeling marine microplastic emissions in Life Cycle Assessment: Characterization factors for biodegradable polymers and their application in a textile case study**

Felicitas Pellengahr^1^*, Elena Corella-Puertas^2^, Valérie Mattelin^3^, Nadim Saadi^2^, Francesca Bertella^1^, Anne-Marie Boulay^2^, Yvonne van der Meer^1^

^1^Aachen Maastricht Institute for Biobased Materials, Maastricht University, Geleen, The Netherlands

^2^Department of Chemical Engineering, CIRAIG, Polytechnique of Montreal, Montreal, QC, Canada

^3^Center for Microbial Ecology and Technology (CMET), Ghent University, 9000 Ghent, Belgium

**Content**

[Supplementary note 1: Fate factors and GLAM characterization factors of the studied polymers 2](#_Toc176538581)

[Supplementary note 2: Conversion of characterization factors to ReCiPe methodology 4](#_Toc176538582)

[Supplementary note 3: Life cycle inventory modeling of the case study 4](#_Toc176538583)

[Supplementary note 3.1: Calculations of the microplastic inventory 9](#_Toc176538584)

[Supplementary note 4: Example of a microscopic image and cell count graphs 10](#_Toc176538585)

[Supplementary note 5: LCIA results of the case study 13](#_Toc176538586)

[Supplementary note 6: Correction factor reasoning 16](#_Toc176538587)

[Literature Cited 17](#_Toc176538588)

# Supplementary note 1: Fate factors and GLAM characterization factors of the studied polymers

Table S1: Fate factors in seawater and sediment compartments of PCL, PLA, and PBSA.

| **Polymer** | **Shape** | **Size (µm)** | **Fate in water (d)** | **Fate in sediments (d)** |
| --- | --- | --- | --- | --- |
| **PCL** | Sphere | 5000 | 5.78E+01 | 1.12E+04 |
|  |  | 1000 | 2.66E+01 | 2.84E+03 |
|  |  | 100 | 1.64E+01 | 2.89E+02 |
|  |  | 10 | 1.06E+01 | 2.01E+01 |
|  |  | 1 | 2.58E+00 | 4.91E-01 |
|  | Fiber | 5000 | 6.72E+01 | 1.38E+04 |
|  |  | 1000 | 2.98E+01 | 3.71E+03 |
|  |  | 100 | 1.69E+01 | 3.89E+02 |
|  |  | 10 | 1.17E+01 | 2.93E+01 |
|  |  | 1 | 3.27E+00 | 8.29E-01 |
|  | Film | 5000 | 8.21E+01 | 1.77E+04 |
|  |  | 1000 | 3.59E+01 | 5.34E+03 |
|  |  | 100 | 1.79E+01 | 5.88E+02 |
|  |  | 10 | 1.29E+01 | 4.85E+01 |
|  |  | 1 | 4.46E+00 | 1.69E+00 |
| **PLA** | Sphere | 5000 | 1.51E+02 | 3.63E+04 |
|  |  | 1000 | 1.03E+02 | 2.33E+04 |
|  |  | 100 | 3.33E+01 | 4.63E+03 |
|  |  | 10 | 1.75E+01 | 5.00E+02 |
|  |  | 1 | 1.25E+01 | 3.98E+01 |
|  | Fiber | 5000 | 1.56E+02 | 3.77E+04 |
|  |  | 1000 | 1.14E+02 | 2.63E+04 |
|  |  | 100 | 3.82E+01 | 5.95E+03 |
|  |  | 10 | 1.82E+01 | 6.68E+02 |
|  |  | 1 | 1.33E+01 | 5.64E+01 |
|  | Film | 5000 | 1.61E+02 | 3.91E+04 |
|  |  | 1000 | 1.28E+02 | 3.01E+04 |
|  |  | 100 | 4.71E+01 | 8.35E+03 |
|  |  | 10 | 1.96E+01 | 1.00E+03 |
|  |  | 1 | 1.43E+01 | 9.01E+01 |
| **PBSA** | Sphere | 5000 | 1.31E+02 | 3.09E+04 |
|  |  | 1000 | 7.14E+01 | 1.49E+04 |
|  |  | 100 | 2.40E+01 | 2.17E+03 |
|  |  | 10 | 1.58E+01 | 2.14E+02 |
|  |  | 1 | 9.53E+00 | 1.35E+01 |
|  | Fiber | 5000 | 1.39E+02 | 3.31E+04 |
|  |  | 1000 | 8.21E+01 | 1.78E+04 |
|  |  | 100 | 2.66E+01 | 2.84E+03 |
|  |  | 10 | 1.64E+01 | 2.89E+02 |
|  |  | 1 | 1.06E+01 | 2.01E+01 |
|  | Film | 5000 | 1.49E+02 | 3.57E+04 |
|  |  | 1000 | 9.79E+01 | 2.20E+04 |
|  |  | 100 | 3.14E+01 | 4.13E+03 |
|  |  | 10 | 1.72E+01 | 4.40E+02 |
|  |  | 1 | 1.21E+01 | 3.41E+01 |

Table S2: GLAM characterization factors (CFs) for PCL, PLA, and PBSA.

| **Polymer** | **Shape** | **Size (µm)** | **GLAM midpoint CF (PAF*year/ kg_emitted_)** | **GLAM endpoint CF (PDF*year/ kg_emitted_)** |
| --- | --- | --- | --- | --- |
| **PCL** | Sphere | 5000 | 5.01E+07 | 6.25E-11 |
|  |  | 1000 | 1.34E+07 | 1.67E-11 |
|  |  | 100 | 1.38E+06 | 1.73E-12 |
|  |  | 10 | 9.55E+04 | 1.19E-13 |
|  |  | 1 | 3.24E+03 | 4.04E-15 |
|  | Fiber | 5000 | 6.09E+07 | 7.59E-11 |
|  |  | 1000 | 1.73E+07 | 2.16E-11 |
|  |  | 100 | 1.87E+06 | 2.33E-12 |
|  |  | 10 | 1.39E+05 | 1.73E-13 |
|  |  | 1 | 5.08E+03 | 6.32E-15 |
|  | Film | 5000 | 7.80E+07 | 9.71E-11 |
|  |  | 1000 | 2.46E+07 | 3.07E-11 |
|  |  | 100 | 2.83E+06 | 3.52E-12 |
|  |  | 10 | 2.29E+05 | 2.85E-13 |
|  |  | 1 | 9.50E+03 | 1.18E-14 |
| **PLA** | Sphere | 5000 | 1.84E+08 | 2.29E-10 |
|  |  | 1000 | 1.25E+08 | 1.55E-10 |
|  |  | 100 | 2.87E+07 | 3.58E-11 |
|  |  | 10 | 3.29E+06 | 4.10E-12 |
|  |  | 1 | 2.79E+05 | 3.48E-13 |
|  | Fiber | 5000 | 1.90E+08 | 2.37E-10 |
|  |  | 1000 | 1.38E+08 | 1.72E-10 |
|  |  | 100 | 3.64E+07 | 4.53E-11 |
|  |  | 10 | 4.38E+06 | 5.46E-12 |
|  |  | 1 | 3.89E+05 | 4.85E-13 |
|  | Film | 5000 | 1.97E+08 | 2.45E-10 |
|  |  | 1000 | 1.56E+08 | 1.94E-10 |
|  |  | 100 | 4.98E+07 | 6.20E-11 |
|  |  | 10 | 6.53E+06 | 8.13E-12 |
|  |  | 1 | 6.13E+05 | 7.63E-13 |
| **PBSA** | Sphere | 5000 | 1.56E+08 | 1.95E-10 |
|  |  | 1000 | 8.10E+07 | 1.01E-10 |
|  |  | 100 | 1.31E+07 | 1.63E-11 |
|  |  | 10 | 1.34E+06 | 1.67E-12 |
|  |  | 1 | 9.44E+04 | 1.18E-13 |
|  | Fiber | 5000 | 1.66E+08 | 2.07E-10 |
|  |  | 1000 | 9.50E+07 | 1.18E-10 |
|  |  | 100 | 1.71E+07 | 2.13E-11 |
|  |  | 10 | 1.81E+06 | 2.25E-12 |
|  |  | 1 | 1.37E+05 | 1.70E-13 |
|  | Film | 5000 | 1.78E+08 | 2.22E-10 |
|  |  | 1000 | 1.15E+08 | 1.43E-10 |
|  |  | 100 | 2.45E+07 | 3.05E-11 |
|  |  | 10 | 2.73E+06 | 3.40E-12 |
|  |  | 1 | 2.25E+05 | 2.81E-13 |

# Supplementary note 2: Conversion of characterization factors to ReCiPe methodology

The characterization factors were primarily developed for the IMPACT World+ methodology (Corella-Puertas et al. 2023). Therefore, they had to be converted to be applicable to the endpoint category unit of damage on ecosystem quality. This was achieved by using the average species density in marine ecosystems of 3.46E-12 species/m^3^ (as proposed for the ReCiPe methodology (Huijbregts et al. 2017)) and the average continental seawater depth of 100 m, according to the USEtox model (Fantke et al. 2017)).

# Supplementary note 3: Life cycle inventory modeling of the case study

The sports shirt production was assumed to occur in China, one of the largest textile producing countries worldwide. It starts with the yarn production, for which PLA granulate is used. The yarn is knitted and dyed, with textile waste going to incineration without energy recovery (as assumed by Horn et al. (2023)). The knitted fabric is cut and assembled into a shirt. The packaged shirt is shipped to the Netherlands where it is used for 52 times. After each use the shirt is washed and occasionally dried (34% of the washing cycles (Sandin et al. 2019)). For the end-of-life, a simplified model was built, taking into account a mixed fate according to latest data on textile waste in the Netherlands. These include the incineration with energy recovery in the Netherlands (55%) and the export to countries such as Pakistan which serves as an example here (European Environmental Agency 2023).

Modeling details are provided in the following tables:

- **Production phase:** Table S3 - Table S7
- **Transport phase:** Table S8
- **Use phase:** Table S9 - Table S11
- **End-of-life phase**: Table S12 - Table S15

Table S3: Life cycle inventory of the yarn production in China, considering the functional unit as described in the manuscript.

| **Activity** | **Quantity** | **Unit** | **Notes** |
| --- | --- | --- | --- |
| *Input* |  |  |  |
| PLA granulate | 0.201 | kg | Ecoinvent 3.9 dataset for PLA |
| Fiber spinning | 0.201 | kg | See Table S4 |
| Yarn spinning | 0.199 | kg | See Table S5 |
| *Output* |  |  |  |
| PLA yarn to knitting | 0.198 | kg |  |
| PLA waste | - | kg | Included in spinning and yarning |

Table S4: Life cycle inventory of the PLA fiber spinning, considering 1 kg of fiber., based on (Sandin et al. 2019) model of melt spinning of polyester.

| **Activity** | **Quantity** | **Unit** | **Notes** |
| --- | --- | --- | --- |
| *Input* |  |  |  |
| Lubricating oil | 0.01 | kg |  |
| Antimony | 0.0002 | kg |  |
| Toluene diisocyanate | 0.0002 | kg |  |
| Electricity | 1.5 | kWh |  |
| Heat | 2.2 | MJ |  |
| *Output* |  |  |  |
| PLA waste | 0.01 | kg | To incineration |

Table S5: Life cycle inventory of the yarn production, based on Sandin et al. 2019, assuming the worst case of yarning PES; per 1 kg of spun yarn

| **Activity** | **Quantity** | **Unit** | **Notes** |
| --- | --- | --- | --- |
| *Input* |  |  |  |
| Electricity | 3.8 | kWh |  |
| Lubricant | 0.0016 | kg |  |
| *Output* |  |  |  |
| PLA waste | 0.005 | kg | To incineration |

Table S6: Life cycle inventory of the knitting and dyeing for one shirt. According to private communication and ecoinvent 3.9.

| **Activity** | **Quantity** | **Unit** | **Notes** |
| --- | --- | --- | --- |
| *Input* |  |  |  |
| Electricity | 0.072 | kWh |  |
| Oil | 0.003 | kg |  |
| Dyeing of knitted fabric | 0.188 | kg | Based on Sandin et al. 2019, adapted to Chinese energy mix |
| *Output* |  |  |  |
| PLA waste | 0.005 | kg | To incineration |

Table S7: Life cycle inventory for garment preparation and assembly of one shirt, based on Moazzem et al. (2018) and Horn et al. (2023).

| **Activity** | **Quantity** | **Unit** | **Notes** |
| --- | --- | --- | --- |
| *Input* |  |  |  |
| Electricity | 0.448 | kWh |  |
| Polyester thread | 0.0006 | kg |  |
| Heat | 0.012 | MJ |  |
| Water | 0.030 | L |  |
| Wicking chemical | 0.002 | kg |  |
| Plastic packaging | 0.002 | kg |  |
| Paper hangtags | 0.002 | kg |  |
| *Output* |  |  |  |
| Wastewater to treatment | 0.030 | L |  |
| PLA waste fabric | 0.005 | kg | To incineration |

Table S8: Life cycle inventory for transport within China and shipping from China to the Netherlands.

| **Activity** | **Quantity** | **Unit** | **Notes** |
| --- | --- | --- | --- |
| *Transport within China by truck* |  |  |  |
| Truck | 0.201 | tkm | Assuming a generic distance of 1000 km |
| *Shipping to the Netherlands* |  |  |  |
| Sea freight transport to Rotterdam | 2.961 | tkm | Based on sea-distances.org |
| Transport from Rotterdam to Appeldoorn | 0.021 | tkm | Exemplary transport distance within NL |

Table S9: Life cycle inventory of the use phase of the functional unit.

| **Activity** | **Quantity** | **Unit** | **Notes** |
| --- | --- | --- | --- |
| *Online order delivery* |  |  | Based on Hischier 2018 |
| Transport – lorry | 0.026 | tkm | Adapted to NL |
| Transport - van | 0.007 | tkm | Adapted to NL |
| *Laundry* |  |  |  |
| Washing | 8.32 | kg | 52 washing cycles according to functional unit; see Table S10 |
| Drying | 2.91 | kg | 35% of washing cycles; see Table S11 |

Table S10: Life cycle inventory of the washing process, based on Sandin et al. 2019, scaled on 1 kg of laundry.

| **Activity** | **Quantity** | **Unit** | **Notes** |
| --- | --- | --- | --- |
| *Input* |  |  |  |
| Water | 6.2 | L |  |
| Detergent | 0.016 | kg | Average detergent |
| Electricity | 0.225 | kWh |  |
| *Output* |  |  |  |
| Wastewater to treatment | 5.2 | L |  |

Table S11: Life cycle inventory of the tumble-drying process, based on Sandin et al. 2019, scaled on 1 kg of laundry.

| **Activity** | **Quantity** | **Unit** | **Notes** |
| --- | --- | --- | --- |
| *Input* |  |  |  |
| Electricity | 0.67 | kWh |  |
| *Output* |  |  |  |
| Wastewater to treatment | 1 | L |  |

Table S12: Life cycle inventory of the PLA textile waste treatment in municipal incineration in the Netherlands, assuming a share of 55% of total waste and allocating 100% of burdens and benefits of the incineration to the shirt’s life cycle (i.e., allocation factor of 1).

| **Activity** | **Quantity** | **Unit** | **Notes** |
| --- | --- | --- | --- |
| *Input* |  |  |  |
| Transport for collection | 0.009 | tkm | Generic transport for collection by truck |
| Shirt incineration | 0.088 | kg | Assuming polyester incineration in municipal incineration plant |
| *Output* |  |  |  |
| Electricity from incineration of PLA, avoided impact | 0.093 | kWh | Electricity efficiency: 0.21; heating value: 18.2 MJ/kg |
| Heat from incineration of PLA, avoided impact | 0.320 | MJ | Heat efficiency: 0.20 |

Table S13: Life cycle inventory of the PLA textile waste exported to Pakistan (serving as an exemplifying case); assuming 0.045 open burning, 0.2655 landfilling, and 0.1395 recycling (allocation factor of 0.5)

| **Activity** | **Quantity** | **Unit** | **Notes** |
| --- | --- | --- | --- |
| *Incineration (open burning)* |  |  |  |
| Transport to Pakistan | 0.007 | kg | See Table S14 |
| Open burning of the shirt | 0.007 | kg |  |
| *Landfilling (open dump)* |  |  |  |
| Transport to Pakistan | 0.042 | kg | See Table S14 |
| Landfilling of the shirt | 0.042 | kg |  |
| *Recycling* |  |  |  |
| Transport to Pakistan | 0.011 | kg | See Table S14 |
| Mechanical recycling of textile | 0.011 | kg | See Table S15 |

Table S14: Life cycle inventory for transport from the Netherlands to Pakistan, per 1 kg of textile.

| **Activity** | **Quantity** | **Unit** | **Notes** |
| --- | --- | --- | --- |
| Truck transport within NL | 0.1 | tkm | Assuming a generic transport distance of 100 km |
| Sea freight transport from NL to South Asian Pakistan Terminals | 11.358 | tkm | According to routescanner.com |

Table S15: Life cycle inventory for mechanical recycling of waste textile, based on Duhoux et al. (2021), per 1 kg of textile.

| **Activity** | **Quantity** | **Unit** | **Notes** |
| --- | --- | --- | --- |
| *Input* |  |  |  |
| Electricity | 0.5 | kWh |  |
| Water | 0.02 | kg |  |
| *Output* |  |  |  |
| Spinnable fibers | 0.25 | kg |  |
| Fluff | 0.075 | kg | Substituting polyester fiber |
| Filling material | 0.3 | kg | Substituting polyurethane foam |

## Supplementary note 3.1: Calculations of the microplastic inventory

We used the Plastic Footprint Network’s recommendations (Plastic Footprint Network 2024) to calculate the plastic emissions of the FU per life cycle stage. Additionally, a worst-case scenario (“high emissions” scenario) was implemented that assumes the emission of the textile waste that is not incinerated in NL. For the worst case, a fragmentation rate of 100% was used, i.e., the macroplastics would fragment completely into microplastics. The emissions per life cycle stage are shown in Table S16.

Table S16: Plastic emissions associated with the FU of one sports shirt throughout its entire life cycle based on the PLP for granulate emissions and PFN for microfiber emissions.

| **Life cycle stage** | **Occurrence of emission** | **Formation** | **Release Rate Ocean** | **Amount (mg) per kg** | **Amount (mg) per jersey** |
| --- | --- | --- | --- | --- | --- |
| Yarn production | CN | Granulate | 1.00 | 1.00E-05 | 2.01E-06 |
| Knitting and dyeing | CN | Microfiber | 0.97 | 2.38E+02 | 4.7E+01 |
| Laundry | NL | Microfiber | 0.07 | 2.54E+02 | 4.07E+01 |
| End-of-life | PK | Microfiber | 1.00 |  | 6.26E+04 |
| Total base case |  |  |  |  | 8.76E+01 |
| Total worst case |  |  |  |  | 6.27E+04 |

# Supplementary note 4: Example of a microscopic image and cell count graphs


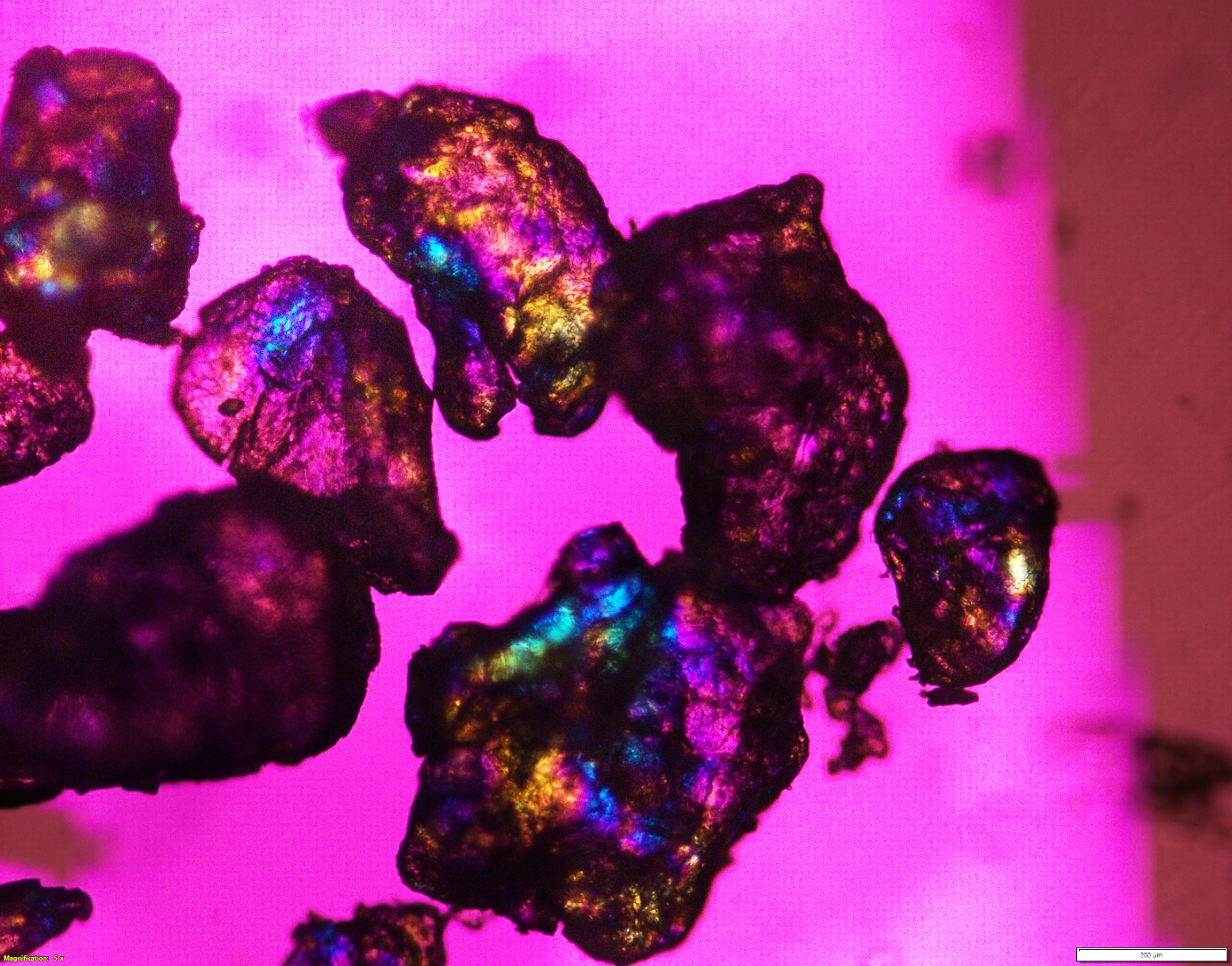


Figure S1: Microscopic image of PLA powder prior to incubation. These images served as a basis for the determination of the size and shape of the particles (magnification: 5x).

Figure S2: Cell count over time of PCL samples. Above: Comparison of all PCL samples, with “abio” indicating the cell count measured for the abiotic sample. Details are shown in the four individual graphs below. For the sample handles see manuscript Table 2.

Figure S3: Cell count over time of PBSA and PLA samples. For the sample handles see manuscript Table 2.

# Supplementary note 5: LCIA results of the case study

Figure S4: Results of the life cycle impact assessment (midpoint impact categories 1-6) of the case study, considering the functional unit as “using a sports shirt weekly over a period of one year in the Netherlands in 2023”.

Figure S5: Results of the life cycle impact assessment of the case study, (midpoint impact categories 7-12), considering the functional unit as “using a sports shirt weekly over a period of one year in the Netherlands in 2023”.

Figure S6: Results of the life cycle impact assessment of the case study (midpoint impact categories 13-18), considering the functional unit as “using a sports shirt weekly over a period of one year in the Netherlands in 2023”.

# Supplementary note 6: Correction factor reasoning

As stated in the manuscript in Section 3, we propose the use of the correction factor $f_{corr}$ (see Eq. (7) in the manuscript) to estimate the surface degradation-related mass loss when only degradation data for macroplastic samples can be found in the literature. This could avoid the underestimation of the degradation time which was discussed in the manuscript.

The correction factor is based on the ratio between the surface-area-to-volume ratios of the micro and the macro particle and aims to correct the order of magnitude of the resulting SSDR. For spheric particles of PCL we found the exponential factor of 2/3 to describe the ratio in the best way. The exponential factor could be influenced by the particle shape and ratio of bulk to surface degradation speed. To further develop the correction factor, the exponential factor needs to be tested for other particle sizes and shapes as well as for other polymers. By employing this correction factor, degradation data from literature that only consider larger particles, could still be used without overestimating the degradation rate and therefore underestimating the residence time and impact of the microplastic particle.

To determine the SSDR of the microplastic, which is necessary for the fate factor, the corrected mass loss would then be used in Eq. (4) of the manuscript. The authors want to emphasize that the altered mass loss does not represent a physically correct figure but serves as a conservative approximation of the actual specific surface degradation rate that would be present for a microplastic particle. An example of the calculation is shown below in the case of PCL grade A. The approach needs to be verified through a greater sample size.

Eq. (8) and (9) (see manuscript) are used to determine the correction factor and the corrected mass loss, considering the data for the PCL granulate:

$$f_{corr, PCL grade A}=\left( \frac{d_{micro}}{d_{macro}} \right)^{\frac{2}{3}}=\left( \frac{132 \mu m}{1751 \mu m} \right)^{\frac{2}{3}}=0.179$$

$${\frac{\Delta m}{m_{0}}}_{corr}=f_{corr}\times{\frac{\Delta m}{m_{0}}}_{macro}=0.179 \times0.26 = 0.047$$

The corrected mass loss is used in Eq. (4) from the manuscript:

$$v_{d, PCLcorrected}=\frac{1}{2}\frac{d_{0}}{t}\left( 1-\sqrt[a]{1-\frac{\Delta m}{m_{0}}} \right)=\frac{1}{2}\frac{1751 \mu m}{182 d}\left( 1-\sqrt[3]{1-0.047} \right)=0.076\frac{\mu m}{d}=27.644 \frac{\mu m}{a}$$

The resulting SSDR of 27.644 µm/a has the same order of magnitude as the SSDR directly obtained through the experiments (31.795 µm/a).

Literature Cited

Corella-Puertas, E., C. Hajjar, J. Lavoie, and A.-M. Boulay. 2023. MarILCA characterization factors for microplastic impacts in life cycle assessment: Physical effects on biota from emissions to aquatic environments. Journal of Cleaner Production **418:**138197.

Duhoux, T., E. Maes, M. Hirschnitz-Garbers, K. Peeters, L. Asscherickx, M. Christis, B. Stubbe, P. Colignon, M. Hinzmann, and A. Sachdeva. 2021. Study on the technical, regulatory, economic and environmental effectiveness of textile fibres recycling, Luxembourg.

European Environmental Agency. 2023. EU exports of used textiles in Europe's circular economy. https://www.eea.europa.eu/publications/eu-exports-of-used-textiles (June 21, 2024).

Fantke, P., M. Bijster, M. Z. Hauschild, M. Huijbregts, O. Jolliet, A. Kounina, V. Magaud, M. Margni, T. E. McKone, R. K. Rosenbaum, D. van de Meent, and R. van Zelm. 2017. USEtox® 2.0 Documentation (Version 1.00). USEtox® Team.

Hischier, R. 2018. Car vs. Packaging—A First, Simple (Environmental) Sustainability Assessment of Our Changing Shopping Behaviour. Sustainability **10:**3061.

Horn, S., K. M. Mölsä, J. Sorvari, H. Tuovila, and P. Heikkilä. 2023. Environmental sustainability assessment of a polyester T-shirt - Comparison of circularity strategies. The Science of the total environment **884:**163821.

Huijbregts, M., Z. Steinmann, P. Elshout, G. Stam, F. Verones, M. Viera, A. Hollander, M. Zijp, and R. van Zelm. 2017. ReCiPe 2016 v1.1 A harmonized life cycle impact assessment method at midpoint and endpoint level, Bilthoven, NL.

Moazzem, S., F. Daver, E. Crossin, and L. Wang. 2018. Assessing environmental impact of textile supply chain using life cycle assessment methodology. The Journal of The Textile Institute **109:**1574–1585.

Plastic Footprint Network. 2024. Assessment Methodology. https://www.plasticfootprint.earth/assessment-methodology/ (November 04, 2024).

Sandin, G., S. Roos, B. Spak, B. Zamani, and G. M. Peters. 2019. Environmental assessment of Swedish clothing consumption.
